# A two-timescale model of plankton–oxygen dynamics predicts formation of oxygen minimum zones and global anoxia

**DOI:** 10.1007/s00285-024-02107-7

**Published:** 2024-05-27

**Authors:** Pranali Roy Chowdhury, Malay Banerjee, Sergei Petrovskii

**Affiliations:** 1 Department of Mathematics and Statistics, Indian Institute of Technology Kanpur, Kanpur, India; 2 School of Computing and Mathematical Sciences, University of Leicester, Leicester LE1 7RH, Leicester, UK; 3 Peoples Friendship University of Russia (RUDN University), 6 Miklukho-Maklaya St, Moscow, Russian Federation 117198

**Keywords:** Slow-fast dynamics, Pattern formation, Extinction, Ocean anoxia, Transients, 35B36, 35Q92, 37N25, 37M05, 92D25, 92D30, 92D40

## Abstract

Decline of the dissolved oxygen in the ocean is a growing concern, as it may eventually lead to global anoxia, an elevated mortality of marine fauna and even a mass extinction. Deoxygenation of the ocean often results in the formation of oxygen minimum zones (OMZ): large domains where the abundance of oxygen is much lower than that in the surrounding ocean environment. Factors and processes resulting in the OMZ formation remain controversial. We consider a conceptual model of coupled plankton–oxygen dynamics that, apart from the plankton growth and the oxygen production by phytoplankton, also accounts for the difference in the timescales for phyto- and zooplankton (making it a “slow-fast system”) and for the implicit effect of upper trophic levels resulting in density dependent (nonlinear) zooplankton mortality. The model is investigated using a combination of analytical techniques and numerical simulations. The slow-fast system is decomposed into its slow and fast subsystems. The critical manifold of the slow-fast system and its stability is then studied by analyzing the bifurcation structure of the fast subsystem. We obtain the canard cycles of the slow-fast system for a range of parameter values. However, the system does not allow for persistent relaxation oscillations; instead, the blowup of the canard cycle results in plankton extinction and oxygen depletion. For the spatially explicit model, the earlier works in this direction did not take into account the density dependent mortality rate of the zooplankton, and thus could exhibit Turing pattern. However, the inclusion of the density dependent mortality into the system can lead to stationary Turing patterns. The dynamics of the system is then studied near the Turing bifurcation threshold. We further consider the effect of the self-movement of the zooplankton along with the turbulent mixing. We show that an initial non-uniform perturbation can lead to the formation of an OMZ, which then grows in size and spreads over space. For a sufficiently large timescale separation, the spread of the OMZ can result in global anoxia.

## Introduction

Plankton is a vital element in the complex marine food webs and biochemical cycles. Phytoplankton is the primary producer standing at the base of the marine food web. As a by-product of the primary production, phytoplankton produces oxygen in the process called photosynthesis. The produced oxygen is then used by marine fauna, e.g. zooplankton and fish. The level of dissolved oxygen is a crucial indicator of the marine ecosystem health, as its depletion may lead to a mass mortality of aquatic species (Heinze et al. 2021; Watson et al. 2017; Wignall and Twitchett 1996). Furthermore, a considerable part of the oxygen produced in the ocean goes to the atmosphere through the ocean surface. It is estimated that around 50–80% of atmospheric oxygen originates in the ocean. Phytoplankton therefore plays a critical role in producing and maintaining oxygen levels needed for survival both of aquatic and terrestrial species (Petsch 2003; Berner 1999).

Over the last few decades, the level of the dissolved oxygen in the ocean has shown a trend to decrease (Schmidtko et al. 2017; Breitburg et al. 2018; Keeling et al. 2010; Oschlies et al. 2018, 2017). This is believed to be a result of the global warming, in particular because warmer water contains less dissolved oxygen (Helm et al. 2011; Schmidtko et al. 2017). Also, the warming leads to a stronger stratification of the upper ocean, which reduces the O_2_ fluxes through the ocean surface (Matear et al. 2000; Bopp et al. 2002). Apart from the above purely physical mechanisms, there can be more subtle effects of the global warming driven by a biological feedback. An increase in the water temperature may slow down the oxygen production by phytoplankton (Jones 1977; Li et al. 1984; Robinson 2000; Hancke and Glud 2004), potentially resulting in a regime shift and a global oxygen depletion (Petrovskii et al. 2017; Sekerci and Petrovskii 2015a, 2018). Ocean anoxia is thought to be the factor that can trigger a mass extinction and this has indeed happened several times during the deep past (Wignall and Twitchett 1996; Erwin 2014; Song et al. 2014; Sudakow et al. 2022). Thus, better understanding of the pathways leading to the global anoxia as well as the identification of possible early signs of the approaching catastrophe are obviously problems of literally vital importance. In turn, it requires a better understanding of the coupled plankton–oxygen dynamics in the ocean and, arguably, mathematical modelling is a powerful research approach to facilitate it.

Mathematical models of plankton dynamics are abundant in the literature, e.g. see (Beltrami 1996; Ascioti et al. 1993; Edwards and Brindley 1996; Steele 1974; Steele and Henderson 1992; Cushing 1975; Truscott and Brindley 1994a, b). Earlier modelling studies of the plankton dynamics were mostly concerned with the temporal and spatio-temporal dynamics of the coupled phytoplankton–zooplankton system (Steele 1978; Steele and Henderson 1992; Medvinsky et al. 2002; Malchow et al. 2000), with a particular focus on plankton patchiness and plankton blooming (Beltrami 1996; Truscott and Brindley 1994b; Scheffer et al. 2000, 1997; Martin 2003). In Scheffer (1991); Petrovskii and Malchow (2000, 2001); Petrovskii et al. (2002); Rinaldi and Muratori (1993), conceptual two- and three-component mathematical models were considered to reveal the role of various internal and external factors and to demonstrate different routes to plankton pattern formation. More recently, there has been growing attention to possible links between the dissolved oxygen, plankton dynamics and the climate change (Charlson et al. 1987; Sarmiento et al. 1998), which facilitated further progress in mathematical modelling of marine ecosystems (Hays et al. 2005). In particular, it was shown in Petrovskii et al. (2017); Sekerci and Petrovskii (2015a, 2015b) that sustainable oxygen production by marine phytoplankton can be severely disrupted by a gradual increase in the average water temperature. In turn, this may eventually lead to a global anoxia and it was argued in Petrovskii (2021) that the observed decrease in the oxygen stock in the ocean (Schmidtko et al. 2017; Breitburg et al. 2018; Battaglia and Joos 2017) and the slow gradual decrease in the amount of atmospheric oxygen (Martin et al. 2017) that has occurred over the last few decades may be early signs of the approaching catastrophe.

Remarkably, the amount of dissolved oxygen does not only change with time, it also depends on space. The spatial distribution of oxygen in the ocean is distinctly heterogeneous (Ito et al. 2017; Richardson and Bendtsen 2017), sometimes resulting in the formation of large stable areas or ‘patches’ where the dissolved oxygen concentration is much lower than the average. Such a patch is referred to as oxygen minimum zone (OMZ) or the dead zone (Breitburg et al. 2018; Watson et al. 2017; Stramma et al. 2008). They were discovered in different parts of the world ocean, e.g. in the subsurface waters of the Arabian Sea and in the eastern boundary upwelling regions of the tropical oceans of California, Peru, and Namibia (Morrison et al. 1999; Stramma et al. 2008). The existence of OMZ has a significant effect on the marine species abundance and the aquatic food chains (Diaz and Rosenberg 2008). There is evidence that some zooplankton species may have a capacity to adapt to oxygen-deficient environments (Wishner et al. 2018). However, a significant drop in the dissolved oxygen level eventually results in the formation of a dead zone, so that the majority of marine life either dies or leaves the area (Diaz and Rosenberg 2008; Watson et al. 2017).

Interestingly, over the last several decades the OMZs have been growing in size. In particular, a rapid expansion in OMZs in the eastern Pacific and northern Indian oceans is well documented (Stramma et al. 2008; Breitburg et al. 2018). Because of the OMZ’s detrimental effect on the corresponding local marine ecosystem, this is becoming a grave concern for ecology and conservation as well as some industries, e.g. fishery. Moreover, there is some theoretical evidence that the OMZ expansion may be an early warning signal of the approaching global anoxia (Alhassan 2023). The expansion of OMZ is thought to be caused by various factors, ultimately linking it to the global warming and to the human interference through the perturbation of the ocean’s biogeochemical cycles, in particular the CO_2_ cycle, although the issue as a whole remains controversial (Keeling et al. 2010; Lenton et al. 2008).

In this study, we address the phenomena of the OMZ formation and growth theoretically based on the earlier conceptual modelling approach that considers the variations (in particular, a decrease) in the dissolved oxygen level as a inherent property of the coupled plankton–oxygen dynamics in the ocean, not necessarily an effect of exogenous factors (Sekerci and Petrovskii 2015a; Petrovskii et al. 2017; Sekerci and Petrovskii 2018). Our updated mathematical model incorporates two important factors that were overlooked in the earlier studies. One such factor is the nonlinear mortality of zooplankton; it takes into account a combined effect of the zooplankton intraspecific competition, cannibalism, and the zooplankton consumption by its predators from a higher trophic level (e.g. fish) (Steele and Henderson 1992; Scheffer 1991). It is well known that the nonlinear mortality rate can change the system’s dynamical properties significantly (Truscott and Brindley 1994b; Bazykin 1998; Chowdhury et al. 2023) but its potential effect on the plankton–oxygen dynamics has never been investigated.

The second factor is the existence of different timescales in the plankton–oxygen dynamics. Indeed, it is a common observation that the zooplankton growth rate is usually much lower than that of phytoplankton. Correspondingly, the typical time (timescale) of changes in the phytoplankton density is considerably shorter (sometimes by an order of magnitude) than that of zooplankton. In this study, we therefore assume that the production of oxygen and the phytoplankton growth, on the one hand, and the zooplankton response, on the other hand, happen on fast and slow timescales, respectively. The presence of different timescales in a dynamical system can make its properties much more complicated, e.g. to bring bifurcations, coexistence of multiple attractors, complex oscillations, long transients and pattern formation that would not be there otherwise (Rinaldi and Muratori 1992; Kuehn 2015; Poggiale et al. 2020; Sadhu 2021; Chowdhury et al. 2023). It is therefore a very relevant question as to how the existence of multiple timescales can modify the plankton–oxygen dynamics, in particular in the context of the OMZ formation and growth.

The paper is organised as follows. In the next section, we describe our mathematical model and investigate its basic properties such as the existence and stability of the steady states. In Sects. 3 and 4, we consider the properties of the nonspatial model to reveal, respectively, the effect of the nonlinear mortality and the different timescales. We identify conditions when the system can undergo a regime shift that may correspond to catastrophic changes in the real world. In Sect. 5, we consider the properties of the spatially explicit system, with a special focus on the dynamical regimes resulting in the pattern formation, in particular those that can be interpreted as the formation and/or expansion of Oxygen Minimum Zones. Finally, in Sect. 6 we summarise and discuss our results.

## The non-spatial system

We consider a conceptual mathematical model that explicitly includes only phytoplankton, zooplankton, and dissolved oxygen. Oxygen is produced by phytoplankton in photosynthesis and consumed by both phytoplankton and zooplankton as needed for their metabolism.

In the zero-dimensional (nonspatial) case, the model is given by the following three equations (Sekerci and Petrovskii 2015a; Petrovskii et al. 2017):

1a $$\begin{aligned} \frac{dc}{dt}&= \frac{Au}{c+1} - \frac{\delta uc}{c+c_2}- \frac{\nu cv}{c+c_3} - c \equiv F(c,u,v), \end{aligned}$$

1b $$\begin{aligned} \frac{du}{dt}&= \left( \frac{B c}{c+c_1} - u \right) u - \frac{u v}{u+h} - \sigma u \equiv G(c,u,v), \end{aligned}$$

1c $$\begin{aligned} \frac{dv}{dt}&= \varepsilon \Big (\frac{\eta c^2}{c^2+{c_4}^2}\frac{uv }{u+h} - \mu _1 v-\mu _2 v^2\Big ) \equiv \varepsilon H(c,u,v), \end{aligned}$$

where $c,\,u$ and *v* denote, respectively, the concentration of dissolved oxygen, phytoplankton, and zooplankton densities in appropriately chosen dimensionless variables (Sekerci and Petrovskii 2015a; Petrovskii et al. 2017) and *t* is dimensionless time. The first term in Eq. (1a) describes the rate of oxygen production in photosynthesis (see (Sekerci and Petrovskii 2015a) for more details) and the second and third terms describe the oxygen consumption by phyto- and zooplankton, respectively, which is assumed to be described by the Monod type kinetics. The first term in Eq. (1b) describes the phytoplankton multiplication; based on earlier work (Steele and Henderson 1981, 1992; Franks 2002), we consider it as the logistic growth. The second term in Eq. (1b) quantifies the phytoplankton grazing by zooplankton (Franks 2002), and the third term describes the phytoplankton natural mortality. In Eq. (1c), the first term in the brackets describes the zooplankton growth (with the food assimilation efficacy being assumed to depend on the level of dissolved oxygen (Sekerci and Petrovskii 2015a)), the second term describes the zooplankton natural mortality and the third (quadratic) term describes a combined effect of the competition and the predation by species from higher trophic levels (not included into the model explicitly) (Truscott and Brindley 1994a). Here parameter *A* is the per capita oxygen production rate, *B* is the per capita phytoplankton growth rate, $\sigma$ and $\mu _1$ are natural mortality rates for phyto- and zooplankton, respectively, $\mu _2$ quantifies the strength of nonlinear zooplankton removal. The meaning of other parameters in Eq. (1) is straightforward. For more details, including biological justification of all terms in the right-hand side of Eq. (1), see (Sekerci and Petrovskii 2015a; Petrovskii et al. 2017).

For the reasons mentioned in the introduction (see Appendix for technical details), we introduce a small parameter $0<\varepsilon <1$ that quantifies the difference in the phyto- and zooplankton characteristic timescales; in most cases below, we will assume $\varepsilon \ll 1$. Note that, compared to the original model proposed in Sekerci and Petrovskii (2015a); Petrovskii et al. (2017), Eq. (1) include two essentially new elements, i.e. the quadratic mortality term for zooplankton and a small parameter (cf. Eq. (1c)), which makes model (1) somewhat more realistic.

### Steady states analysis

To explore the dynamics of the temporal model, we study all possible equilibrium points (steady states) of the system (1) and their stability. The system has a total extinction state given by $E_0=(0,0,0).$ To study the dynamics of the system (1) around $E_0$ we linearize around $E_0$ and obtain the Jacobian matrix

2 $$\begin{aligned} J_{E_0}= \begin{pmatrix} -1&{}&{}A&{}&{}0\\ 0&{}&{}-\sigma &{}&{}0\\ 0&{}&{}0&{}&{}-\varepsilon \mu _1 \end{pmatrix}. \end{aligned}$$

All the eigenvalues of the above matrix $J_{E_0}$ are real and negative. Therefore, the total extinction state $E_0$ is always stable. In Sekerci and Petrovskii (2015a) the authors showed that under some parametric restrictions, the system can have two zooplankton free equilibria. Since the introduction of the quadratic term in (1c) does not affect the zooplankton free equilibrium states, as the zooplankton free state is the form $(\bar{c},\bar{u},0)$ where $\bar{c}$ is the root of the quartic equation

3 $$\begin{aligned}{} & {} \bar{c}^4-(\delta (\sigma -B)-(c_1+c_2+1))\bar{c}^3 \nonumber \\ {}{} & {} \quad - (A(B-\sigma )+(\delta \sigma -c_2-1)c_1-B\delta +\delta \sigma -c_2)\bar{c}^2 \nonumber \\ {}{} & {} \quad -(((B-\sigma )c_2-\sigma c_1)A+\delta \sigma c_1-c_1c_2)\bar{c} + A\sigma c_1c_2=0, \end{aligned}$$

and

4 $$\begin{aligned} \bar{u} = \frac{\bar{c}(B-\sigma )-c_1\sigma }{\bar{c}+c_1}. \end{aligned}$$

**Fig. 1:**
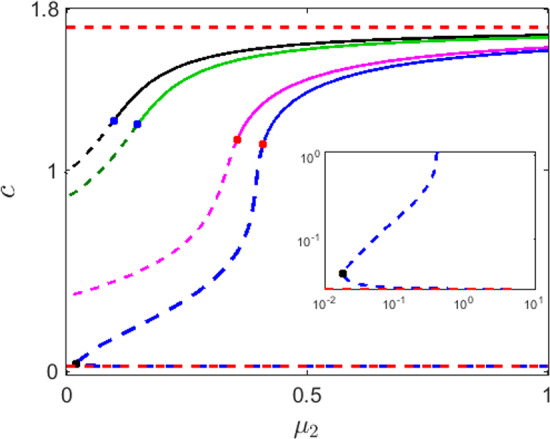
The plot of *c*-component of the coexistence equilibrium for the parameter values given in (5) as a function of parameter $\mu _2$ for $\varepsilon =1$ and a few different values of $\mu _1$: $\mu _1=0$ (blue), $\mu _1=0.05$ (magenta), $\mu _1=0.25$ (green), and $\mu _1=0.3$ (black). The black dot represents the saddle-node bifurcation threshold $(c^{\textrm{SL}}, \mu _2^{\textrm{SL}}).$ The inset shows the nature of the equilibrium branch near the saddle-node bifurcation threshold in log-log scale. The red horizontal lines represent the *c*-component of the two zooplankton-free equilibrium points. The red dots on the equilibrium branches represent subcritical Hopf bifurcation threshold, while the blue dots on the equilibrium branches represents supercritical Hopf bifurcation threshold

Since the equation (3) is a fourth-order polynomial, analytical determination of the equilibrium points is nearly impossible. We choose suitable numerical values of the parameters to obtain feasible zooplankton free equilibrium states. Throughout the paper, we fix the parameter values at

5 $$\begin{aligned}{} & {} A = 4,\,B=3,\,\sigma = 0.1,\, c_1 = 0.7,\, c_2 = 1,\, c_3 = 1,\nonumber \\{} & {} \quad c_4 = 1,\, \eta = 0.7,\, \delta = 1,\, {\nu = 0.01,}\, h = 0.1. \end{aligned}$$

and suitably vary $\mu _1,\,\mu _2,$ and $\varepsilon .$ For all the values of $\mu _1$ and $\mu _2$ and parameters fixed at (5) the two feasible zooplankton free equilibrium points are given by $E_1=(0.0258,0.0067,0)$ and $E_2=(1.712,2.029,0).$ Among the two zooplankton free equilibrium points, one is always a saddle point while the other can be either stable or unstable depending on the parameter values. For our choice of parameter values (5) both $E_1$ and $E_2$ are unstable (saddle) with a 2-dimension stable manifold and a 1-dimensional unstable manifold. We plot the *c*-component of $E_1$ and $E_2$ in Fig. 1 with red broken lines. Note the lines are horizontal, because the corresponding steady state values do not depend on $\mu _1$ or $\mu _2$, as is obvious from Eqs. (3–4). The system does not possess any other feasible boundary equilibria in this parametric regime. Note that a nontrivial oxygen free equilibrium is not possible in our model (which agrees with biological reasons). Indeed, setting $c\equiv 0$ in Eq. (1a) immediately leads to $u\equiv 0$, which in turn leads to $z\equiv 0$.

The coexistence equilibrium of the system (1) is denoted by $E_*=(c_*,u_*,v_*)$ where

$$\begin{aligned} v_*=\frac{1}{\mu _2}\Big (\frac{\eta c_*^2}{c_*^2+c_4^2}\frac{u_*}{u_*+h}-\mu _1\Big ) \end{aligned}$$

and $c_*,\,u_*$ can be obtained by simultaneously solving the quartic and quadratic equations respectively

$$\begin{aligned}{} & {} c_*^4 + (\delta u_* + \nu v_*+ c_2 + c_3+1)c_*^3 - ((A-\delta (1+c_3))u_* - (1+c_2)\nu v_* \nonumber \\ {}{} & {} \quad - (c_2(1+c_3)+c_3))c_*^2 \\{} & {} \quad - (Au_*(c_2+c_3)-(\delta u_*c_3+\nu v_*c_2+c_2c_3))c - Au_*c_2c_3 = 0,\\{} & {} \quad u_*^2(c_*+c_1) - ((B-h-\sigma )c_*-(h+\sigma )c_1)u_* \nonumber \\ {}{} & {} \quad - (Bh-h\sigma -v_*)c_* + (h\sigma +v_*)c_1 = 0. \end{aligned}$$

Because of the complexity of the simultaneous algebraic equations we obtain the coexistence equilibrium points using numerical simulations. The parameter values are fixed at (5) and we choose $\mu _1,\ \mu _2$ as the bifurcation parameters. Note that for $\mu _1>0,\,\mu _2=0,$ the model was rigorously studied in Sekerci and Petrovskii (2015a). However, we deal with other cases in this work. For $\mu _1=0,\,\mu _2>0,$ we found that the system (1) has two coexistence equilibrium points which disappears via saddle-node bifurcation for sufficiently smaller values of $\mu _2$. If we denote the saddle-node bifurcation threshold by $\mu _2^{\textrm{SL}},$ then for $\mu _2>\mu _2^{\textrm{SL}}$ the system has two coexistence equilibrium points and for $\mu _2<\mu _2^{\textrm{SL}}$ there are no feasible coexistence equilibrium. For $\mu _1,\,\mu _2>0,$ the system has a unique coexistence equilibrium throughout the parametric regime of $\mu _2,$ whenever they are feasible. The number of coexistence equilibrium points and their stability is determined by the combination of the parameters $\mu _1$ and $\mu _2.$ The Jacobian matrix evaluated at the coexistence equilibrium $E_*$ is

6 $$\begin{aligned} J_{E_*}=\begin{pmatrix} J_{11}&{}&{}J_{12}&{}&{}J_{13}\\ J_{21}&{}&{}J_{22}&{}&{}J_{23}\\ J_{31}&{}&{}J_{32}&{}&{}J_{33}\\ \end{pmatrix} \end{aligned}$$

and the corresponding characteristic equation is given by

$$\begin{aligned} \lambda ^3+p_2\lambda ^2+p_1\lambda +p_0=0. \end{aligned}$$

The coefficients are

$$\begin{aligned} p_2=-\textrm{tr}(J_{E_*}),\,\,p_1=J_{11}^{[1]}+J_{22}^{[2]}+J_{33}^{[3]},\,\,p_0=-\det (J_{E_*}) \end{aligned}$$

where $J_{ij}$ is the (*i*, *j*)-th entry of $J_{E_*},$ and $J_{ii}^{[i]}$ is the cofactor of $J_{ii}.$ By Routh–Hurwitz conditions the coexistence equilibrium point $E_*$ is stable if the following conditions hold

$$\begin{aligned} p_0>0,\,p_2>0\,\,\text {and}\,\,p_1p_2>p_0. \end{aligned}$$

To observe the change in system’s dynamics with the introduction of intraspecific competition among the zooplankton, we choose $\mu _2$ to be the bifurcation parameter. Thus keeping all the parameters fixed, $p_1,\,p_2,\,p_0$ are functions of $\mu _2$ only. The coexistence equilibrium $E_*$ loses its stability and exhibits oscillatory dynamics through Hopf bifurcation at $\mu _2^{\textrm{H}},$ which is obtained by solving

$$\begin{aligned} p_1(\mu _2^{\textrm{H}})p_2(\mu _2^{\textrm{H}}) = p_0(\mu _2^{\textrm{H}}). \end{aligned}$$

This expression is not mathematically tractable, thus we numerically obtain the Hopf bifurcation threshold $\mu _2^{\textrm{H}}= 0.35405$ (upto five decimal place) for the parameter values (5), and $\mu _1=0.05,\,\varepsilon =1.$ The Hopf bifurcation can be either supercritical or subcritical depending on the magnitude of the parameters $\mu _1$ and $\mu _2.$ For a fixed $\mu _1,$ with an increase in the intraspecific competition (as quantified by $\mu _2$), the equilibrium state of the system changes its stability from unstable to stable.

The broken line in Fig. 1 represents the unstable branch of the equilibrium and the continuous line represents the stable branch. The red and blue dots on the equilibrium branch are the subcritical and supercritical Hopf bifurcation thresholds, respectively. For a relatively higher value of $\mu _2,$ both the extinction state $E_0$ and the coexistence state $E_*$ is stable, and the system exhibits bi-stability. For $\mu _1=0$ the lower branch of the coexistence equilibrium is saddle and it acts as a separatrix between the basin of attraction of $E_*$ and $E_0.$ However, for $\mu _1>0,$ their basins are separated by the unstable manifold of the saddle boundary equilibrium point. For a fixed $\mu _2$ we observe an increase in the oxygen concentration with an increasing mortality rate of the zooplankton (see Fig. 1). Therefore, an increase in the zooplankton linear mortality rate (as quantified by $\mu _1$) and an increase in the nonlinear mortality rate (due to the intraspecific competition and/or predation by higher trophic levels (Steele and Henderson 1992), as quantified by $\mu _2$) lead to an increase in the stable oxygen level.

## Local and global dynamics

**Fig. 2:**
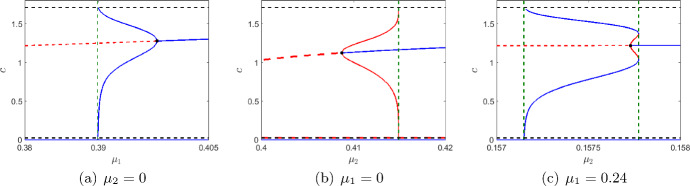
Bifurcation diagram of the system (1) at the parameter values (5) for different combination of $\mu _1$ and $\mu _2.$ The stable equilibrium and limit cycles are marked in blue. The unstable cycle and coexistence equilibrium are marked in red (continuous and broken respectively). The *c*-component of the boundary equilibrium points is shown by broken black lines. The global bifurcation thresholds are marked in vertical broken green line. The black dot represents the Hopf bifurcation threshold (subcritical and supercritical)

The dynamics of the system (1) is determined by its local and global bifurcation structure. However, any comprehensive analytical analysis of the bifurcations is hardly possible for this model due to its algebraic complexity. Instead, we choose $\mu _1$ and $\mu _2$ as bifurcation parameters and obtain two corresponding one parametric bifurcation diagrams; see Fig. 2. These diagrams readily reveal the two Hopf bifurcation scenarios of the coexistence equilibrium. We further show the possibility of the heteroclinic bifurcation and saddle-node bifurcation of limit cycles.

In Fig. 2, the Hopf bifurcation thresholds (supercritical and subcritical) are marked by black dots and the thresholds for the global bifurcations (heteroclinic and saddle-node bifurcation of limit cycles) are marked by green vertical broken lines. The stable and unstable cycles (and equilibrium) are shown in blue and red colour, respectively. We choose $\mu _1$ or $\mu _2$ as bifurcation parameters to observe the change in the system’s dynamics. Note that, the zooplankton free equilibrium points $E_1$ and $E_2$ are independent of the bifurcation parameters. However, the stability of these equilibrium points depend on $\mu _1.$ For our investigation, we choose all other parameters as in (5), and observe that $E_1$ is unstable for all feasible values of $\mu _1.$ Whereas, $E_2$ is unstable for $\mu _1 \in [0,0.497].$ Therefore, both $E_1$ and $E_2$ are unstable for the set of parameter values chosen in this work. We plot the $\bar{c}$-components of the unstable zooplankton free equilibrium points of the form $(\bar{c},\bar{u},0)$ in a black broken line.

In the absence of intra-specific competition among the zooplankton and neglecting the effect of higher trophical levels (that is, for $\mu _2=0$), the system has a unique coexistence equilibrium, which loses its stability through Hopf bifurcation at $\mu _1^{\textrm{H}}=0.398$ [(considering all the other parameters are fixed at (5)]. The Hopf bifurcation is supercritical as the first Lyapunov coefficient is $l_1= -0.358 (<0)$. The unique coexistence equilibrium point is stable for $\mu _1>\mu _1^{\textrm{H}}$ and unstable for $\mu _1<\mu _1^{\textrm{H}}.$ A small amplitude stable limit cycle originates from $\mu _1^{\textrm{H}}.$ The amplitude of the stable cycle increases with decreasing $\mu _1$ till it hits the boundary equilibrium points and disappears via a heteroclinic bifurcation (shown in Fig. 2 by the green lines). Beyond this threshold, the coexistence equilibrium is unstable and the extinction state $E_0$ is the only global attractor. On decreasing the mortality rate from $\mu _1^\textrm{H}$ the concentration of the zooplankton increases. This increases the oxygen consumption by zooplankton and also decreases phytoplankton production such that beyond a threshold the system collapse.

On the contrary, if we consider the case where the zooplankton mortality is primarily due to the combined effect of the competition and predation by species from higher trophic levels, then one could neglect the natural mortality (Steele and Henderson 1992), so that $\mu _1=0$. Using other parameter values as in (5), the Hopf bifurcation occurs at $\mu _2^{\textrm{H}} = 0.408.$ Since the first Lyapunov coefficient is $l_1=1.065(>0),$ the Hopf bifurcation is subcritical. Among the two coexistence equilibria as obtained in Fig. 1, one is always saddle, the other is unstable for $\mu _2<\mu _2^{\textrm{H}}$ and stable for $\mu _2>\mu _2^{\textrm{H}}.$ An unstable cycle emerges from the subcritical Hopf bifurcation, increases in size in a narrow domain for $\mu _2>\mu _2^{\textrm{H}},$ till it disappears via global bifurcation (see Fig. 2b). Beyond this, the stable extinction state $E_0$ coexists with a stable coexistence equilibrium. We now choose $\mu _1=0.24.$ Then, the subcritical Hopf bifurcation occurs at $\mu _2^{\textrm{H}}=0.1577.$ Here the unstable cycle that emerged through Hopf bifurcation is surrounded by a stable limit cycle. The stable and unstable cycle exists in a narrow range of $\mu _2$ and collides at saddle-node bifurcation of limit cycle ($\mu _2=0.1578).$ On the other hand, for $\mu _2<\mu _2^{\textrm{H}},$ the unstable equilibrium is surrounded by a stable limit cycle. The amplitude of the stable cycle increases and disappears through heteroclinic bifurcation at $\mu _2=0.15715$ (see Fig. 2c).

## Slow and fast dynamics

The system (1) for $0<\varepsilon \ll 1$ is referred to as the singularly perturbed system. The timescale parameter $\varepsilon$ signifies a clear distinction between two timescales: slow and fast. The change in the zooplankton density occurs at a much slower rate as compared to the change in oxygen and phytoplankton density. We denote the time ‘*t*’ in system (1) as the fast time and the system (1) is with respect to the fast timescale.

Introducing the slow timescale $\tau =\varepsilon t,$ we obtain the following system with respect to slow time:

7 $$\begin{aligned} \begin{aligned} \varepsilon \frac{dc}{d\tau }&= \frac{Au}{c+1} - \frac{\delta uc}{c+c_2}- \frac{\nu cv}{c+c_3} - c \equiv F(c,u,v), \\ \varepsilon \frac{du}{d\tau }&= \left( \frac{B c}{c+c_1} - u \right) u - \frac{u v}{u+h} - \sigma u \equiv G(c,u,v), \\ \frac{dv}{d\tau }&= \Big (\frac{\eta c^2}{c^2+{c_4}^2}\frac{uv }{u+h} - \mu _1 v-\mu _2 v^2\Big ) \equiv H(c,u,v), \end{aligned} \end{aligned}$$

We analyze the above slow and fast systems with the help of geometric singular perturbation theory (Kuehn 2015; Fenichel 1979). The basic idea behind this approach was to decompose the slow and fast systems in its limiting systems (i.e for $\varepsilon =0$) and study the dynamics of the respective subsystems. In the singular limit $\varepsilon \rightarrow 0,$ we obtain the fast subsystem (oxygen-phytoplankton) of system (1) as follows

8 $$\begin{aligned} \begin{aligned} \frac{dc}{dt}&= \frac{Au}{c+1} - \frac{\delta uc}{c+c_2}- \frac{\nu cv_0}{c+c_3} - c, \\ \frac{du}{dt}&= \left( \frac{B c}{c+c_1} - u \right) u - \frac{u v_0}{u+h} - \sigma u, \\ \end{aligned} \end{aligned}$$

with $v=v_0$ (constant zooplankton density). Also, letting $\varepsilon =0,$ in (7) we obtain the slow subsystem or the reduced system as

9 $$\begin{aligned} F(c,u,v) =0,\,\, G(c,u,v) =0,\,\, \frac{dv}{d\tau } = H(c,u,v), \end{aligned}$$

where *F*, *G*, *H* are given above.

### Critical manifold

The set

$$\begin{aligned} C_0 = \{(c,u,v)\in \mathbb {R}^3: F(c,u,v)=0=G(c,u,v) \}, \end{aligned}$$

is called critical manifold, which is the collection of all equilibrium points or curves of the fast subsystem. It can be divided into two parts: trivial critical manifold $C_0^0$ and non-trivial critical manifold $C_0^1$ such that $C_0=C_0^0\cup C_0^1.$ The trivial critical manifold is given by the line

$$\begin{aligned} C_0^0 = \{(c,u,v)\in \mathbb {R}^3: c=0,\,u=0,\,v\in \mathbb {R}\} \end{aligned}$$

and the non-trivial critical manifold $C_0^1$ is the curve of intersection of two surfaces given by

$$\begin{aligned} v=(u+h)\Big (\frac{Bc}{c+c_1}-u-\sigma \Big ),\,\,v=\frac{(c+c_3)}{\nu }\Big (\frac{Au}{c+1} - \frac{\delta uc}{c+c_2}-c\Big ). \end{aligned}$$

Because of the complexity of the functions *F* and *G*, it is difficult to explicitly obtain the non-trivial critical manifold analytically. Hence, we analyze the set of all equilibrium points of the fast subsystem (8) for an arbitrary $v_0 \in \mathbb {R}_+,$ by studying the position of the isoclines. From (8), we obtain the oxygen isocline and the phytoplankton isocline as follows for a fixed $v_0$ as

$$\begin{aligned}{} & {} F(c,u,v_0)=0 \implies u = \frac{c(1+c)(c+c_2)(c+c_3+\nu v_0)}{(c+c_3)(Ac+Ac_2-c\delta (1+c))} \\{} & {} G(c,u,v_0)=0 \implies c = \frac{c_1(v_0+u^2+u\sigma +h(u+\sigma ))}{B u - u^2 - v_0 + h (B - u - \sigma ) - u \sigma }. \end{aligned}$$

**Fig. 3:**
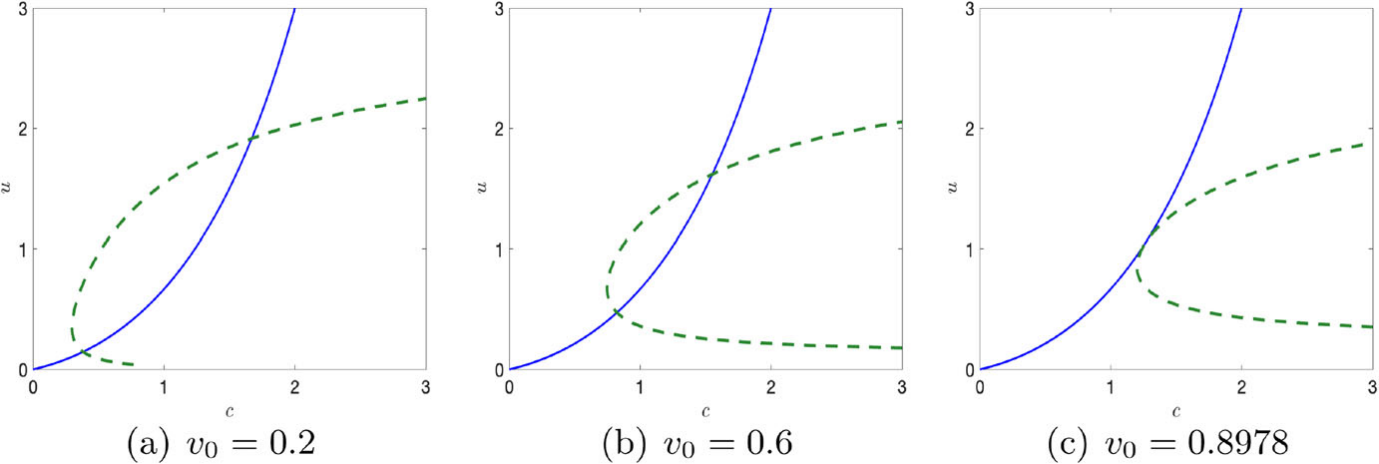
Relative position of the oxygen (blue) and phytoplankton (green broken) nullcline for different values of $v_0.$ The other parameters are kept fixed at $A = 4,\,B=3,\,\sigma = 0.1,\, c_1 = 0.7,\, c_2 = 1,\, c_3 = 1,\,\delta = 1,\,\nu = 0.01,\, h = 0.1$

The change in the position of the nullclines for different values of $v_0$ is shown in Fig. 3. From the geometric properties of the nullclines as shown in the figure, it can be seen that there exists at most two interior equilibrium points. With a gradual increase of $v_0$, the two interior equilibria disappears via a saddle-node bifurcation. We denote this bifurcation point as $(c_s,u_s).$ The Jacobian matrix of the fast subsystem evaluated at $(c_s,u_s)$ is given by

10 $$\begin{aligned} \mathcal {J}_{sub} = \begin{pmatrix} 1 - \frac{A u_s}{(1 + c_s)^2} - \frac{c_2 u_s \delta }{(c_s + c_2)^2} - \frac{c_3 v_0 \nu }{(c_s + c_3)^2}&{}&{}\frac{A}{1+c_s}-\frac{c_s \delta }{c_s + c_2}\\ \frac{B c_1 u_s}{(c_s + c_1)^2}&{}&{}\frac{Bc_s}{c_s+c_1}-\frac{hv_0}{(h+u_s)^2}-2u_s-\sigma \end{pmatrix}. \end{aligned}$$

At the saddle-node bifurcation point we have $\det (\mathcal {J}_{sub})=0.$ Therefore, for all the parameters fixed at (5) and solving simultaneously the following equations

$$\begin{aligned} F(c,u,v_0)=0,\,G(c,u,v_0)=0,\,\det (\mathcal {J}_{sub})=0 \end{aligned}$$

**Fig. 4:**
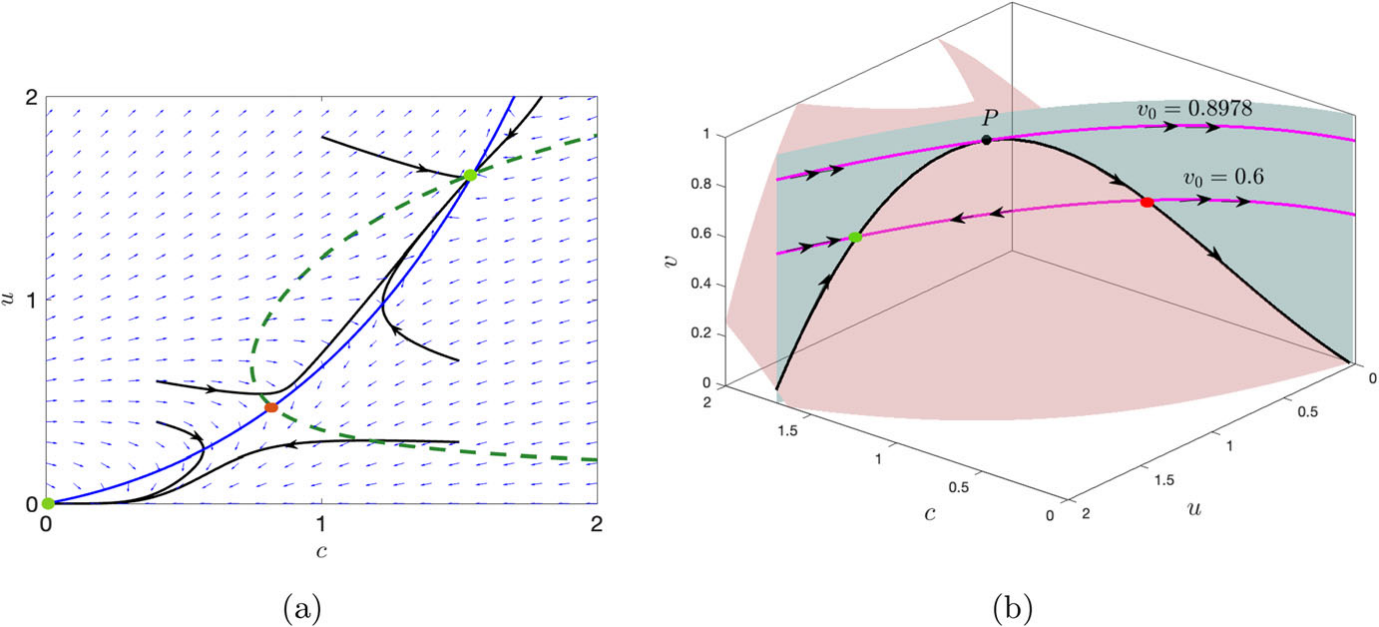
**a** The stability of the equilibrium points of the fast subsystem is shown for $v_0=0.6.$ The small blue arrows represent the direction of vector field in *cu* plane. The Oxygen and phytoplankton nullclines are shown in blue solid line and green broken line, respectively. The trajectories (black) for six different initial conditions are shown. **b** The critical manifold (black) is plotted for the parameter values (5). Corresponding values of $v_0$ is plotted in magenta. The fold point *P*(1.2653, 1.0522, 0.8978) is shown in black dot. The stable and unstable equilibrium of the fast subsystem for a fixed value of *v* is shown in green and red dots respectively. Double and single arrows represent the fast and slow flow respectively. The surfaces $F = 0$ and $G = 0$ are shaded in green and pink, respectively

we obtain the saddle-node bifurcation point $(c_s,u_s,v_s)$ as

$$\begin{aligned} c_s=1.26531,\,u_s=1.05229,\,v_s=0.89784. \end{aligned}$$

Now, collecting the interior equilibrium points of the fast subsystem (8) for all the values of $v_0$ we numerically obtain the critical manifold (black) in the three dimensional space as shown in Fig. 4. Comparing with Fig. 3, we further plot the corresponding values of $v_0$ in magenta color. This shows that the saddle-node bifurcation point of the fast subsystem (8) corresponds to a fold point of the full system (7). We denote this fold point of the full system by *P*; for the given parameter values $P=(1.26531,1.05229,0.89784).$

#### Stability analysis

To determine the stability of the trivial manifold, we evaluate the Jacobian matrix $\mathcal {J}$ along $C_0^0$ and thus obtain

11 $$\begin{aligned} \mathcal {J}_{C^0_0}=\begin{pmatrix} -1-\frac{\nu v}{c_3}&{}&{}A\\ 0&{}&{}-\frac{v}{h}-\sigma \end{pmatrix}. \end{aligned}$$

Since all the parameters involved in the system are positive and $v>0$, all the eigenvalues are negative and given by

$$\begin{aligned} -1-\frac{\nu v}{c_3}<0,\,\,\,-\frac{v}{h}-\sigma <0, \end{aligned}$$

for $v\ge 0.$ Thus the trivial manifold $C_0^0$ is stable for $v\ge 0.$ This implies that the extinction state (0, 0) of the fast subsystem is always stable. As we have shown in Fig. 3, the fast subsystem (8) admits at most two interior equilibrium points say $\tilde{E}_1=(\tilde{c_1},\tilde{u_1})$ and $\tilde{E}_2=(\tilde{c_2},\tilde{u_2})$ such that $\tilde{u_1}<\tilde{u_2}.$ Among the two interior equilibrium points, $\tilde{E}_1$ is always a saddle (hence unstable) and $\tilde{E}_2$ is stable. This can be seen by plotting the direction of the flow $(\frac{dc}{dt},\frac{du}{dt})$ and also tracking the trajectories for different initial points. We have shown this for a particular value of $v_0=0.6$ in Fig. 4a. Therefore, for $v_0 \in \mathbb {R}_+$, collecting all the equilibrium points of the form $(\tilde{c_1},\tilde{u_1})$ such that $\tilde{u_1}<u_s$ form the repelling part of the non-trivial critical manifold, and the part of the critical manifold where $\tilde{u}_2>u_s$ forms the attracting part of the critical manifold. We denote it by $C^{1r}_0$ and $C^{1a}_0,$ respectively, and these two branches are separated by the fold point *P*.

Fenichel’s theorem (Fenichel 1979) state that the normally hyperbolic attracting and repelling sub-manifolds, $C_0^{1a}$ and $C^{1r}_0$ respectively, obtained for $\varepsilon =0,$ perturb to locally invariant attracting and repelling sub-manifolds $C^{1a}_{\varepsilon }$ and $C^{1r}_{\varepsilon }$ respectively, for $\varepsilon >0.$ Therefore, the dynamics of the full system (1) or (7) can be approximated by studying the dynamics of the subsystems obtained for $\varepsilon =0.$ The fast flow is thus obtained on the plane $v=v_0$ and is shown by double arrows in Fig. 4b and the slow flow on the critical manifold $C_0^1$ is shown by the single arrow. The reduced dynamics is given by the slow subsystem (9). The direction of the slow flow is determined by the sign of *H*(*c*, *u*, *v*) at the fold point *P*. Since $H(c_s,u_s,v_s)=0.001859 (>0),$ and *H* is a smooth function, thus in a small neighborhood of the fold point the direction of the slow flow is towards the fold point. We differentiate $F(c,u,v)=0$ and $G(c,u,v)=0$ implicitly with respect to $`\tau$’ along the critical manifold, and obtain the dynamics on the critical manifold. This is governed by the following system of equations

12 $$\begin{aligned} \begin{aligned} \frac{dc}{d\tau } = -\frac{F_vG_u-F_uG_v}{F_cG_u-F_uG_c}H,\,\, \frac{du}{d\tau } = -\frac{F_vG_c-F_cG_v}{G_cF_u-G_uF_c}H,\,\, \frac{dv}{d\tau } = ~~H, \end{aligned} \end{aligned}$$

with suitable initial condition $(c_0,u_0,v_0)\in C_0^1.$ The slow flow has a singularity whenever $G_cF_u-G_uF_c=0,$ which holds at the fold point *P*. Thus, the solution blows up at this point. Whenever $F_vG_u-F_uG_v\ne 0$ or $F_vG_c-F_cG_v\ne 0,$ the fold point is called the jump point, and the trajectory jumps from the proximity of the fold point to another attracting critical manifold. However, when both $F_vG_u-F_uG_v=0$ and $F_vG_c-F_cG_v=0,$ the fold point is called canard point. At this point, the trajectory can pass through the proximity of the fold point and follow the repelling manifold for *O*(1) time.

**Fig. 5:**
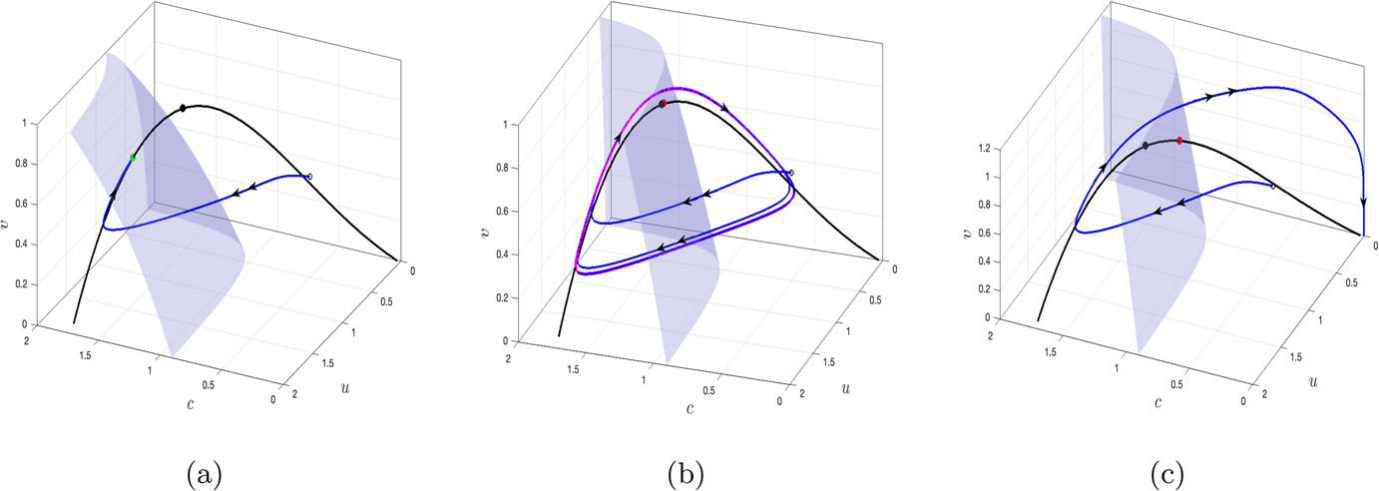
The dynamics of the full system is shown for three cases; **a** when the coexisting equilibrium lies on $C^{1a}_0,$ **b** within a very small neighborhood of the fold point, **c** $C^{1r}_0$. Green and red dots represent the stable and unstable coexisting equilibrium point of the full system (1). Single and double arrows represent the slow and fast flows of the system, respectively. The surface $H=0$ is shown in (shaded) blue

The full system (1) admits an equilibrium point when the surface $H(c,u,v)=0$ intersects with the non-trivial critical manifold $C^1_0.$ For the range of parameters considered in this manuscript, the surface $H=0$ intersects the non-trivial critical manifold at an unique point, either on $C^{1a}_0$ or $C^{1r}_0$ or with the fold point *P*. The dynamics of the full system is shown along with the fast and slow flows when the coexisting equilibrium is stable (cf. Fig. 5a), unstable and the system exhibits canard cycles (Fig. 5b), when the equilibrium is unstable and lies away from the fold point (cf. Fig. 5c). The coexisting equilibrium loses its stability and exhibits oscillatory dynamics through Hopf bifurcation (supercritical or subcritical), which we discussed in the previous section. In a classical slow-fast setting, the small cycles originating from Hopf bifurcation bifurcates to canard cycles (with or without head) and further to relaxation oscillation, thus exhibiting canard explosion (Krupa and Szmolyan 2001). These cycles are composed of slow and fast segments, where the slow flow occurs along both attracting and repelling sub-manifold of the critical manifold. It exhibits fast flow when the trajectory jumps to another stable portion of the critical manifold. However, for the system (1), small canard cycles (without head) emerge from Hopf bifurcation (Fig. 5c). The amplitude of the cycles increases in a narrow parametric range, eventually leading to complete extinction. We prove this fact in the following theorem.

##### Theorem 4.1

Assume the fold point *P* is a canard point for $\varepsilon >0.$ Then the system (1) has small amplitude canard cycles (without head) originating from Hopf bifurcation but there does not exist any relaxation oscillation.

##### Proof

In the slow-fast setting, we denote the Hopf bifurcation as singular Hopf bifurcation since the eigenvalues of the Jacobian matrix of the system (1) evaluated at $E_*$ has purely imaginary complex eigenvalue of the form

$$\begin{aligned} \lambda _{1,2} = \pm ~i \omega (\mu _2^{\textrm{H}},\varepsilon ) \end{aligned}$$

such that $\lim _{\varepsilon \rightarrow 0} \omega (\mu _2^{\textrm{H}},\varepsilon )=0.$ The singular Hopf bifurcation occurs at $O(\varepsilon )$ from the fold point P. We assume $F_vG_u-F_uG_v=0$ and $F_vG_c-F_cG_v=0$ such that the fold point is the canard point. The small limit cycle originating from Hopf bifurcation grows in size through a sequence of canard cycles. With the decrease in $\varepsilon ,$ the amplitude of the cycle increases, and after a certain threshold, the trajectory jumps from the vicinity of the fold point *P* close to $C_0^0.$ The equilibrium point $E_0$ lies on the trivial critical manifold. The eigenvalues of the Jacobian matrix $J_{E_0}$ are $-1,\,-\sigma ,\,\,-\varepsilon \mu _1$ and the corresponding eigenvectors are

$$\begin{aligned} \left( 1,0,0\right) ,\,\left( 1,\frac{1-\sigma }{A},0\right) ,\,\ \text {and}\,\ \left( 0,0,1\right) . \end{aligned}$$

Therefore the critical manifold $C_0^0$ coincides with the eigenvector (0, 0, 1). Thus, any trajectory on $C_0^0$ converges to $E_0.$ We cannot construct any singular orbit consisting of concatenated slow segments on $C_0^1$ and $C_0^0,$ and fast fibers while leaving the respective manifolds. Hence, the global return mechanism, which is necessary for the existence of classical relaxation oscillation, fails as all the trajectories converge to the stable equilibrium $E_0$. $\square$

**Fig. 6:**
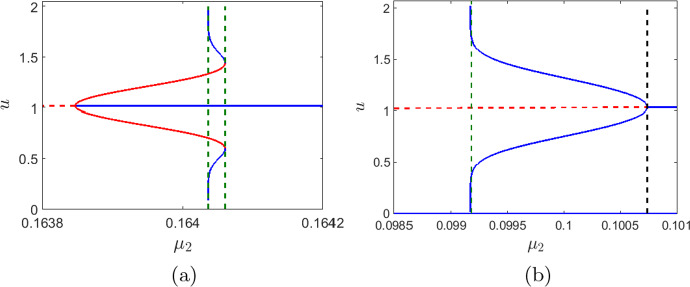
The change in the amplitude of the canard cycle emerging from the singular Hopf bifurcation with varying $\mu _2$ is shown for **a** $\mu _1=0.24,\,\varepsilon =0.5,$ and **b** $\mu _1=0.3,\,\varepsilon =0.5.$ The blue lines show the steady state value of of the coexistence equilibrium when it is stable and the maximum and minimum amplitude of the stable canard cycle when the equilibrium is unstable. The horizontal red (broken) line shows the steady state value of the coexistence equilibrium when it is unstable. The vertical black (broken) line marks the singular Hopf bifurcation threshold (occurring at $\mu _2=0.1007$) and the vertical green (broken) line at $\mu _2=0.099171$ indicate the threshold for the system collapse (plankton extinction and oxygen depletion). Other parameters of the system are given in (5)

We illustrate this phenomenon with the help of a numerical example in Fig. 6. We use the parameter values as in (5) along with $\varepsilon =0.5$. Consider a hypothetical value $\mu _1=0.24.$ The effect of $\varepsilon$ on the dynamics of the system can be observed if we compare the Fig. 6a with Fig. 2c. The singular Hopf bifurcation occurs at $\mu _2^{\textrm{H}}=0.1638$ is subcritical ($l_1=0.037$). Small unstable canard cycles emerge from the canard point *P* (1.24, 1.01, 0.89), which grows in size with a slight increase in $\mu _2$ (Fig. 6a). A large amplitude stable cycle emerges due to a heteroclinic bifurcation that coexists with the stable equilibrium, separated by an unstable canard cycle. We observe that the size of the stable cycle shrinks in an extremely narrow parameter interval and disappears at a saddle node bifurcation of limit cycles.

**Fig. 7:**
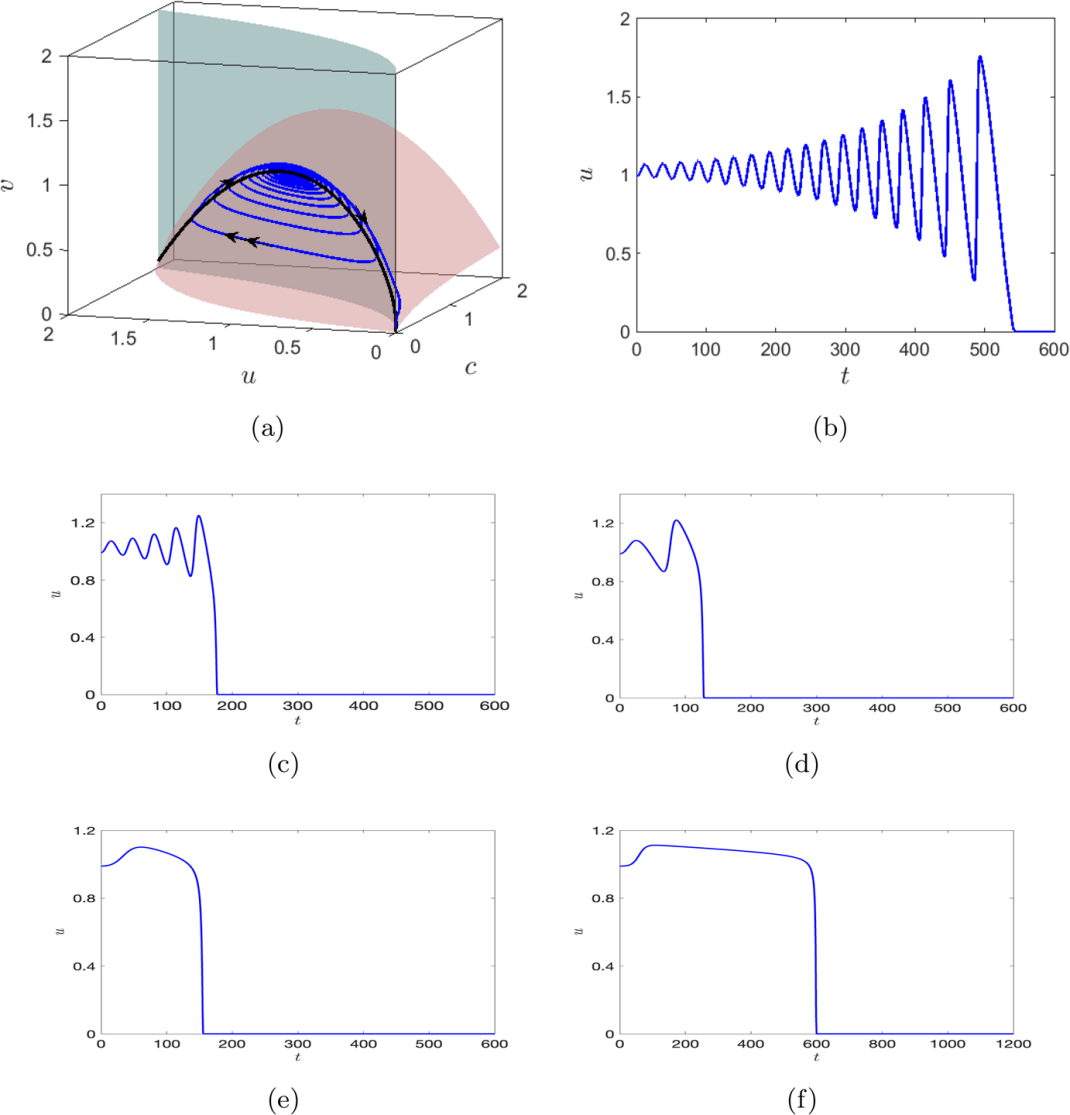
**a** A trajectory (blue) converging to the origin (extinction) after oscillations of increasing amplitude obtained for $\mu _1=0.3,\,\mu _2 = 0.09917$ and $\varepsilon =0.5.$ The other system parameters are mentioned in equation (5). The surfaces $F=0$ and $G=0$ are shaded in green and brown, respectively. The black curve on the intersection of these surfaces is the critical manifold $C^1_0.$ The single and double arrows represent slow and fast motion, respectively. **b** The corresponding dependence of the phytoplankton density on time for $\varepsilon =0.5.$ The time series plot of the phytoplankton density for **c** $\varepsilon =0.3$, **d** $\varepsilon =0.1$, **e** $\varepsilon =0.01$, **f** $\varepsilon =0.001.$

We now consider $\mu _1=0.3$ and $\varepsilon =0.5,$ then the singular Hopf bifurcation occurs at $\mu _2^{\textrm{H}} = 0.1007.$ The first Lyapunov coefficient is $l_1=-1.1818$, hence the singular Hopf bifurcation is supercritical. From the canard point *P* (1.255, 1.035, 0.898),  small stable canard cycles originate. We show the change in the amplitude of the canard cycles with decreasing values of $\mu _2$ in Fig. 6b. This depicts that the system becomes unstable with a decrease in the strength of the intra-specific competition among the zooplankton. The transition from the stable, steady state to the oxygen-free state, indicating complete population collapse, takes place in an extremely narrow interval of the rate of intraspecific competition. That is, for $\mu _2 \in (0.099171,0.1007).$ At $\mu _2=0.09917,$ when the size of the limit cycle explodes, the trajectory converges to the origin, which is illustrated in Fig. 7a. The time series of the trajectory is shown in Fig. 7b. This implies that the system cannot further sustain any large amplitude oscillations. The rise in the amplitude of the phytoplankton level beyond a threshold can act as an indicator of population collapse. The system can therefore be driven to total extinction by pushing it far enough to reach the fold point. We fix $\mu _2=0.099171$ and study the effects of the timescale separation in the oscillatory dynamics of the system. We observe that as we increase the timescale separation from 0.5,  the transient oscillations observed in Fig. 7a decreases (Fig. 7c). That is for $\varepsilon =0.1$ the concentration of phytoplankton declines to zero after one oscillation for $t\in [100,200]$ (Fig. 7d). By decreasing $\varepsilon$ to 0.01, we observe that the system stays at an intermediate state for a certain time before the sudden collapse which occurs between $t\in [100,200]$ (Fig. 7e). Further, for $\varepsilon$ sufficiently small, that is for $\varepsilon =0.001,$ the transient oscillation further decreases but the tendency of the system to stay at an intermediate state increases (Fig. 7f). Therefore as $\varepsilon$ approaches zero we observe a change in the transient dynamics of the system, from oscillatory to non-oscillatory. Moreover, as $\varepsilon \rightarrow 0,$ the trajectory of the system spends a considerable amount of time on the attracting part of the critical manifold, thereby increasing the transient time. It seems that the system might settle down at a steady state around $u=1,$ but as we simulate the system for longer time, we observe that the population collapses at around $t=600.$

## The spatial system

In the real world ocean environment, the spatial distribution of both plankton and dissolved oxygen is remarkably heterogeneous, sometimes showing the variability by an order of magnitude or even more (Steele 1974; Levin 1990; Martin 2003; Ito et al. 2017; Richardson and Bendtsen 2017). Correspondingly, in this section, we consider a spatially extended model (1) where the oxygen concentration and the phytoplankton and zooplankton densities vary with both time and space. The ocean system is three-dimensional; however, in this paper, for the sake of simplicity, we only consider one horizontal spatial dimension. We regard it as the position along the ocean surface. In terms of ocean observations, it corresponds to a transect across the study area.

Trying to keep the model as simple as possible, we avoid using explicit dependence on the vertical dimension (i.e. the depth). Correspondingly, we use the well-mixed layer approximation (Steele and Henderson 1981, 1992; Franks 2002) to assume that the vertical distribution of plankton and oxygen is approximately uniform within the photic (upper) ocean layer where most of photosynthetic oxygen production takes place.

The transport of any substance in the ocean takes place primarily due to the water movement. The movement in the horizontal direction occurs either due to an ocean current or marine turbulence (or their combination). Here we chose to focus on the effect of unbiased (isotropic) movement, hence taking into account only the effect of turbulence, which we describe as the turbulent diffusion quantified by a certain diffusion coefficient (Monin and Yaglom 1971; Okubo 1980).

We, therefore, arrive at the following equations:

13 $$\begin{aligned} \begin{aligned} \frac{\partial c}{dt}&= ~~D_c\frac{\partial ^2{c}}{\partial {x}^2} +\frac{Au}{c+1} - \frac{\delta uc}{c+c_2}- \frac{\nu cv}{c+c_3} - c, \\ \frac{\partial u}{dt}&= ~~D_u\frac{\partial ^2{u}}{\partial {x}^2} +\left( \frac{B c}{c+c_1} - u \right) u - \frac{u v}{u+h} - \sigma u, \\ \frac{\partial v}{dt}&= ~~D_v\frac{\partial ^2{v}}{\partial {x}^2} +\varepsilon \Big (\frac{\eta c^2}{c^2+{c_4}^2}\frac{uv }{u+h} - \mu _1v-\mu _2 v^2\Big ). \end{aligned} \end{aligned}$$

Here $c(x,t),\,u(x,t),$ and *v*(*x*, *t*) are, respectively, the oxygen concentration and the phytoplankton and zooplankton densities at the (horizontal) location *x* and time *t*; $D_c,\,D_u$ and $D_v$ denote the diffusion coefficients for oxygen, phytoplankton, and zooplankton. Note that phytoplankton and the dissolved oxygen can be regarded as a ‘passive substance’, i.e. their spatial transport is entirely determined by the water flows; hence $D_c=D_u=D_T$ where $D_T$ is the turbulent diffusion coefficient. However, zooplankton has a certain ability to self-motion. Combined with the effect of turbulent mixing, it can result in a value of $D_v\ne D_T$. Whether $D_v$ is larger or smaller, then depends on the zooplankton movement pattern. In case zooplankton movement is entirely random (e.g. can be regarded as Brownian motion), then one can expect that $D_v>D_T$. In case zooplankton exhibits a homing behavior, then it is likely that $D_v<D_T$.

Along with the temporal parameters, we now non-dimensionalize the space as $\tilde{x}=\frac{x}{\sqrt{D_c}}$. Removing the tilde for the simplicity of the notation, we obtain the following dimensionless spatial model:

14 $$\begin{aligned} \begin{aligned} \frac{\partial c}{dt}&= ~~\frac{\partial ^2{c}}{\partial {x}^2} +\frac{Au}{c+1} - \frac{\delta uc}{c+c_2}- \frac{\nu cv}{c+c_3} - c, \\ \frac{\partial u}{dt}&= ~~\frac{\partial ^2{u}}{\partial {x}^2} +\left( \frac{B c}{c+c_1} - u \right) u - \frac{u v}{u+h} - \sigma u, \\ \frac{\partial v}{dt}&= ~~D\frac{\partial ^2{v}}{\partial {x}^2} +\varepsilon \Big (\frac{\eta c^2}{c^2+{c_4}^2}\frac{uv }{u+h} - \mu _1v-\mu _2 v^2\Big ), \end{aligned} \end{aligned}$$

where $D = \frac{D_v}{D_c}$. Equations (14) are considered inside the spatial domain $\Omega =\{x\in (0,L)\}$ where *L* is thus the length of the domain.

Equations (14) must be complemented with the initial conditions, which we consider in the following form:

15 $$\begin{aligned} c(x,0){=}\left\{ \begin{array}{ll} {c_*{+}0.1}, &{} |x{-}L/2|{<}10\\ c_*, &{} \text {otherwise} \\ \end{array}\right. ,\,\ u(x,0){=}\left\{ \begin{array}{ll} u_*{+}0.2, &{} |x{-}L/2|{<}10\\ u_*, &{} \text {otherwise} \\ \end{array}\right. ,\nonumber \\ \end{aligned}$$

$$\begin{aligned} v(x,0)\,= v_*,\quad \forall x \in \Omega . \end{aligned}$$

That is, at $t=0$ the steady state densities are perturbed within a small area at the center of the domain $\Omega$.

For the boundary conditions, we consider the zero-flux conditions:

16 $$\begin{aligned} c_x(0,t)\,=\,c_x(L,t)= u_x(0,t)\,=\,u_x(L,t)\,=\,v_x(0,t)\,=\,v_x(L,t)\,=\,0,\,\,t\,>\,0.\nonumber \\ \end{aligned}$$

The model is solved numerically with $L=500.$ We use the Euler method for the temporal part and three points central difference scheme for the diffusion part with $\Delta x=1$ and $\Delta t=0.01.$

### Turing instability

To study the spatial distribution of oxygen and plankton, we start our analysis in a neighborhood of a homogeneous steady-state solution of (14). Time-independent or a steady state solution (*c*(*x*), *u*(*x*), *v*(*x*)) of the system (14)–(16) satisfies the following system of equations

17 $$\begin{aligned} \begin{aligned}&\frac{\partial ^2{c}}{\partial {x}^2} +\frac{Au}{c+1} - \frac{\delta uc}{c+c_2}- \frac{\nu cv}{c+c_3} - c =0, \\&\frac{\partial ^2{u}}{\partial {x}^2} +\left( \frac{B c}{c+c_1} - u \right) u - \frac{u v}{u+h} - \sigma u =0, \\&D\frac{\partial ^2{v}}{\partial {x}^2} +\varepsilon \Big (\frac{\eta c^2}{c^2+{c_4}^2}\frac{uv }{u+h} - \mu _1v-\mu _2 v^2\Big ) =0. \end{aligned} \end{aligned}$$

The coexistence equilibrium $E_*$ of system (1) corresponds to a homogeneous steady state solution of the above system. To study the dynamics of the above system near the homogeneous steady-state solution $E_*,$ we give small heterogenous perturbation as

18 $$\begin{aligned} c(x,t)\!=\!c_*\!+\!\xi _1 e^{\lambda t}\cos kx,\,u(x,t)\!=\!u_*\!+\!\xi _2 e^{\lambda t}\cos kx,\,v(x,t)\!=\!v_*+\xi _3 e^{\lambda t}\cos kx \nonumber \\ \end{aligned}$$

with $0<\xi _1,\xi _2,\xi _3\ll 1.$ The parameter *k* is the wavenumber of the eigenfunction, and $\lambda$ is the eigenvalue determining the temporal growth of the corresponding $k{\textrm{th}}$ mode. We obtain the linearized system as

19 $$\begin{aligned} \textbf{Z}_t = J_{E_*}\textbf{Z} + \mathcal {D}\Delta \textbf{Z} \end{aligned}$$

where $\textbf{Z}\equiv (z_1,z_2,z_3),$ $\mathcal {D}\equiv \textrm{diag}(1,1,D).$ For the non-trivial solution of the above system (19), the eigenvalues $\lambda$ are determined by the roots of the characteristic polynomial $\det (\lambda I -J_{E_*} + \mathcal {D}k^2)=0,$ which is written explicitly as

20 $$\begin{aligned} \lambda ^3+p_2(k^2)\lambda ^2+p_1(k^2)\lambda +p_0(k^2)=0, \end{aligned}$$

where

21 $$\begin{aligned} p_2(k^2)= & {} ~~(2+D)k^2-\textrm{tr}(J_{E_*}),\nonumber \\ p_1(k^2)= & {} ~~(1+2D)k^4 -((J_{22}+J_{33})+(J_{11}+J_{33})+D(J_{11}+J_{22}))k^2\nonumber \\ {}{} & {} \quad + (J_{11}^{[1]}+J_{22}^{[2]}+J_{33}^{[3]}),\nonumber \\ p_0(k^2)= & {} ~~ D k^6 - ((J_{11} + J_{22})D + J_{33}) k^4 + ( J_{11}^{[1]} + J_{22}^{[2]} + J_{33}^{[3]}D) k^2 - \det (J_{E_*}),\nonumber \\ \end{aligned}$$

and $J_{ij}$ and $J_{ii}^{[i]}$ are the same as obtained during the analysis of the temporal part. We, therefore, obtain the necessary and sufficient conditions for Turing instability as

22 $$\begin{aligned} \begin{aligned} p_2(0)>0,\,\,p_0(0)>0,\,\,p_1(0)p_2(0)>p_0(0)\,\,\text {and}\,\,p_0(k^2)<0,\,\,\text {for some}\,\,k. \end{aligned} \end{aligned}$$

Therefore, the Turing instability occurs at a critical wave number, $k=k_T,$ where $p_0(k^2)$ achieves a local minimum and $p_0(k_T^2)=0.$ This gives

23 $$\begin{aligned} \begin{aligned} k_T^2 =&~~ \frac{J_{11}+J_{22}}{3} + \frac{1}{3D} (J_{33} + \sqrt{\Lambda }) \end{aligned} \end{aligned}$$

where

$$\begin{aligned}{} & {} \Lambda = \left( J_{11}^2+J_{22}^2-J_{11}J_{22}+3J_{12}J_{21}\right) D^2 \\ {}{} & {} \quad + D\left( 3J_{13}J_{31}+3J_{23}J_{32}-J_{11}J_{33}-J_{22}J_{33}\right) + J_{33}^2, \end{aligned}$$

and $J_{ij}$ are the elements of the Jacobian matrix, cf. Eq. (6). Because of the complexity of the expression and the large number of parameters involved, the critical wave number $k_T$ corresponding to the Turing bifurcation has to be computed numerically. For a feasible $k_T$, the model describes the formation of spatial patterns, as is shown below.

### Impact of diffusion on oxygen minimum zone

We now look into the spatio-temporal dynamics of the system (14) with the initial conditions (15). Our goal is to reveal typical dynamical regimes (in particular, pattern formation scenarios, if any) for parameters inside and outside of the Turing domain. Within the pure Turing domain all the Turing instability conditions as discussed above holds. However, in a Turing–Hopf domain, the homogeneous steady state is unstable under both temporal and spatio-temporal perturbations. We fix the parameter values as in (5) and let $\mu _1=0,\,\mu _2=0.41$ and consider different values of the diffusivity ratio *D*.

**Fig. 8:**
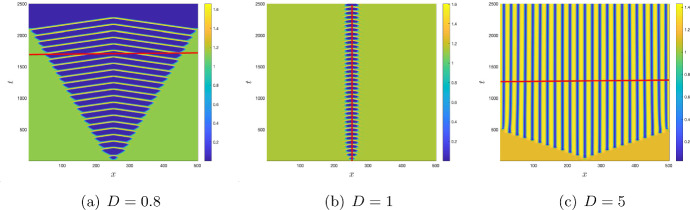
Transition of spatio-temporal dynamics of oxygen from non-Turing (panels **a** and **b**) to Turing (panel **c**) pattern formation for $\mu _1=0,\, \mu _2=0.41,\,\varepsilon =1$ and different values of *D*. All other parameters are given in (5); the corresponding steady state value $c_*\approx 1.2$. The auxiliary red lines help to reveal the properties the oxygen distribution of oxygen at a given moment of time (as in panels **a** and **c**) or at a given location in space (as in panel **b**); see details in the text

Figure 8 shows typical patterns in the distribution of oxygen for different diffusivity rates. Here Fig. 8a–c are obtained, respectively, for parameters outside and inside the Turing instability domain. We notice that, in all three cases, the evolution of the initial conditions soon leads to the formation of a patch where the oxygen concentration is much lower than its steady state value. We interpret this dynamics as the formation of an OMZ. Further evolution of the emerging OMZ can be significantly different depending on *D*. When the diffusivity ratio is small, i.e. $D<1,$ the OMZ created at the early stage grows with time and eventually spreads over the entire domain. Interestingly, the growing OMZ has a fine structure. A closer look at the dynamics shown in Fig. 8a reveals that, at any time *t* during the transient stage of the OMZ expansion, it consists of three or four subdomains with very low oxygen level separated by narrow spatial intervals where the oxygen level is larger than its steady state value $c_*$. This fine structure disappears after the expanding OMZ hits the domain boundaries; at a later time the oxygen level is low over the entire domain, which can be interpreted as the global anoxia.

An increase in the diffusivity ratio to $D=1$ results in a qualitative change in the dynamics; see Fig. 8b. In this case, the OMZ formed at an early stage of the system dynamics show almost no spatial growth and remain localised around the centre of the domain. At any spatial position inside the OMZ, the oxygen level distinctly oscillates with time between a very low level (approximately $0.1c_*$) to a high level (of about $1.3c_*$).

A further increase in *D* leads to another qualitative change in the dynamical pattern. Figure 8c shows the results obtained for $D=5$. In this case, the system satisfies the Turing instability condition with the critical wavenumber $k_T^2 = 0.1095.$ The evolution of the initial conditions leads to the formation, inside a certain subdomain, of a periodic spatial pattern where low oxygen patches alternate with high oxygen patches. The subdomain containing this periodic structure grows with time and eventually occupies the whole domain, so that at a large time the periodic spatial distribution becomes stationary.

**Fig. 9:**
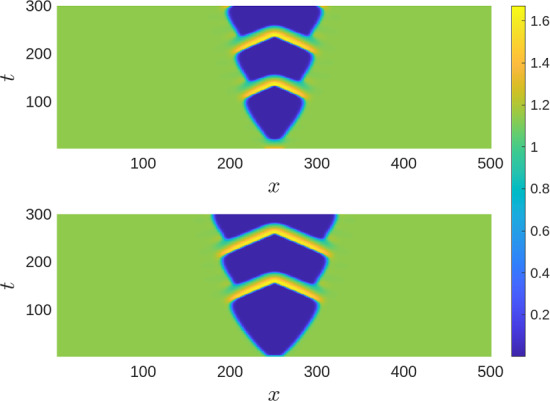
(Top) Zoomed plot of Fig. 8a, which is obtained using the initial condition (15). (Bottom) The initial conditions are of the form (15) but with $c(x,0) = c_*-0.5$ and $u(x,0) = u_*-0.2$ for $|x-\frac{L}{2}|<10$. All the parameters are same as in Fig. 8a. The modified initial conditions therefore result in the formation of the OMZ of a larger size

We note here that the pattern of the OMZ formation and spread is relatively robust to the initial conditions. For instance, if at $t=0$ the spatial distribution of oxygen is perturbed along with that of phytoplankton, the emerging patterns are similar to the ones shown in Fig. 8. One example is shown in Fig. 9. In this case, the initial conditions (15) are slightly modified, so that, at the center of the domain both *c*(*x*, 0) and *u*(*x*, 0) are less than their steady state values. It is readily seen that the top and the bottom of Fig. 9 show very similar patterns, with the only difference that the spatial size of the emerging OMZ becomes larger for the modified initial conditions.

### Impact of timescale separation on oxygen minimum zone

**Fig. 10:**
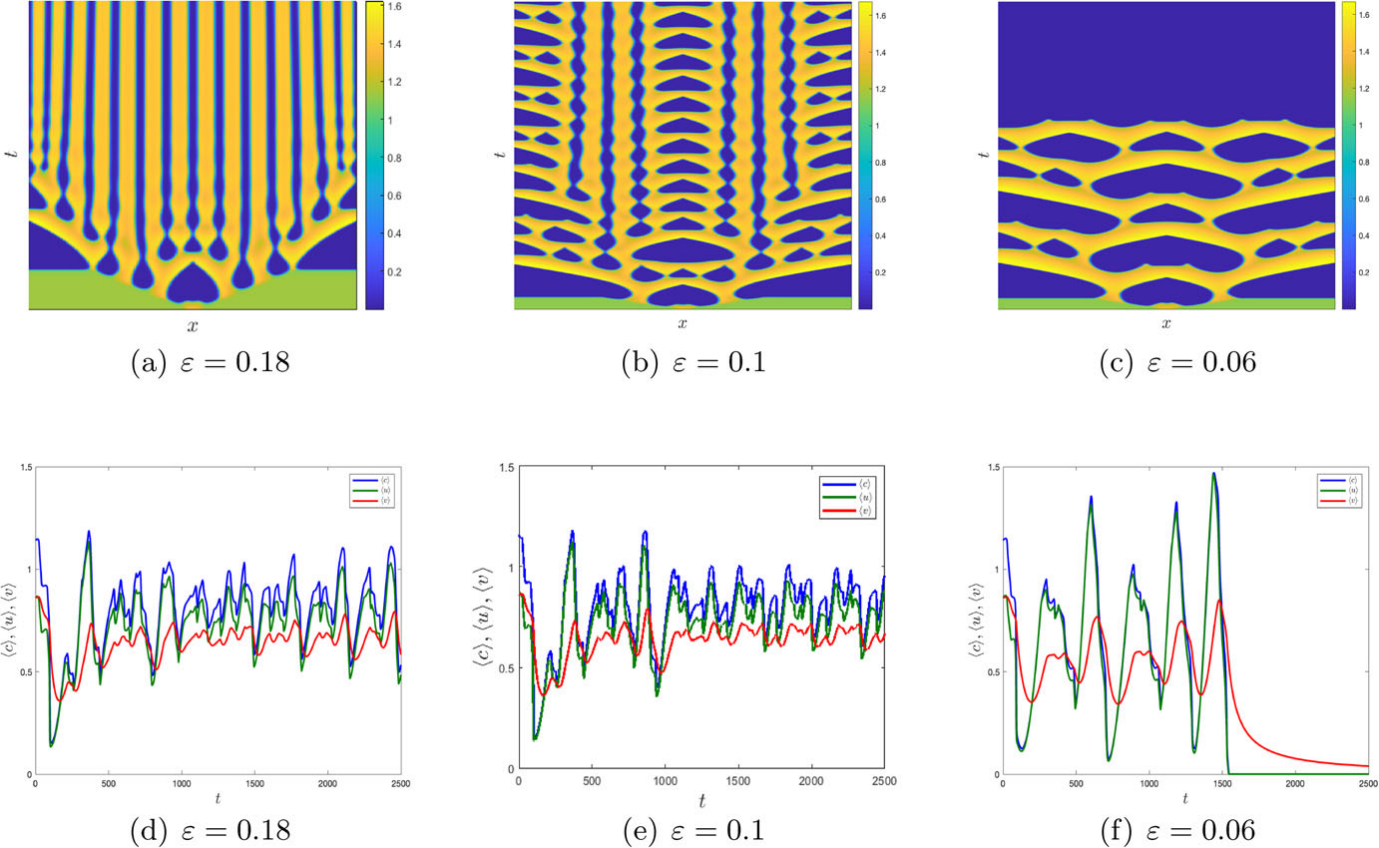
**a**–**c** Spatial distribution of oxygen; **d**–**f** spatial average density of oxygen, phytoplankton, and zooplankton for the parameter values (5) with $\mu _1=0,\,\mu _2=0.41,$ and $D=5.$

From the mathematical analysis in the Sect. 5.1, we obtain that the critical wavenumber $k_T$ for Turing instability depends on the timescale separation $\varepsilon$, as the elements $J_{31}$, $J_{32}$ and $J_{33}$ of the Jacobian matrix in Eq. (23) depend on $\varepsilon$. Thus, one can expect that the boundaries of the parameter ranges where the Turing and the Turing–Hopf instability occur (resulting in pattern formation) can shift with a decrease in $\varepsilon$, i.e. with an increase in the timescale separation. Numerical simulations confirm that this is indeed the case. Having fixed $\mu _1=0,\,\mu _2=0.41$, $D=5$ and other parameters as in (5) and only varying $\varepsilon$, we obtain stationary patterns in both the Turing domain and in the Turing–Hopf domain for $\varepsilon \ge 0.18.$ A typical pattern is shown in Fig. 8c. With a decrease in $\varepsilon$, the emerging stationary pattern has a similar nature of alternating patches of high and low oxygen level as for $\varepsilon =1$ (cf. Fig. 8c) but the size of the patches becomes larger; e.g. see Fig. 10a. Also, the emergence of the stationary periodic pattern is preceded by rather long transient dynamics when the oxygen concentration and the plankton densities exhibit irregular oscillations (see Fig. 10d).

With a further decrease in $\varepsilon$, the dynamics becomes qualitatively different. The emerging pattern is not spatially periodic any more; see Fig. 10b. Apart from the large OMZ formed around the centre of the domain at the early stage of system’s dynamics, there are two large OMZs at the sides of the domain. At a later time, these patches of low oxygen level break to a number of smaller patches of variable size. The dynamics is not becoming stationary at any time as the patches keep changing their size (and some of them also their location). The dependence of spatially average densities is distinctly irregular (see Fig. 10e) suggesting chaotic dynamics. This kind of dynamic pattern is observed for $0.07<\varepsilon <0.18$.

With a further decrease in $\varepsilon$ (below $\varepsilon =0.07$), the system’s dynamics undergo a regime shift. For $\varepsilon <0.07$, the transient apparently chaotic dynamics only last for a finite time. After a sufficiently long time, the system experiences a catastrophic change when over a short transition time the oxygen concentration fast drops to a very small value (and eventually to zero) over the entire spatial domain. An example of such regime shift is shown in Fig. 10c,f (obtained for $\varepsilon =0.06$). The entire spatial domain becomes a dead zone (with low or no oxygen), which can be interpreted as the global anoxia. Along with oxygen, the phyto- and zooplankton densities go to zero as well (after several irregular oscillations of increasing amplitude, cf. Fig. 10f), obviously signifying their extinction.

**Fig. 11:**
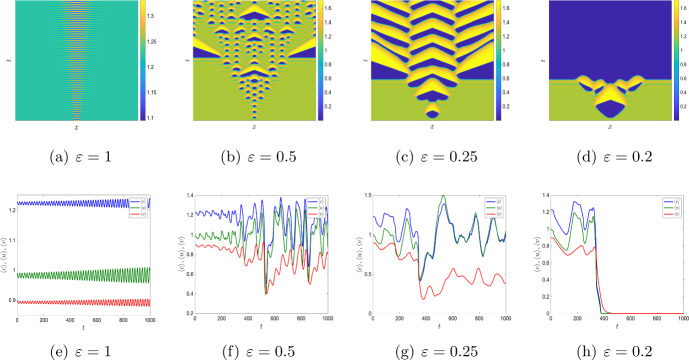
**a**–**d** Transition of spatial distribution of oxygen and **e**–**h** spatial average density of oxygen, phytoplankton, and zooplankton for the parameter values (5) and $\mu _1=0.24,\,\mu _2=0.1575,\,D=1$ and different values of $\varepsilon .$

For both $\mu _1>0$ and $\mu _2>0$, the system’s dynamics becomes different and exhibits a somewhat greater variety of dynamical regimes. As one example, Fig. 11a,e shows the spatiotemporal dynamics for $\mu _1=0.24,\,\mu _2=0.1575$, $D=\varepsilon =1$ and other parameters the same as in Fig. 8b,e. It is readily seen that, in this particular case, the evolution of the initial condition does not lead to formation of OMZ. It only leads to small fluctuations in the oxygen level and plankton densities around the location of the initial perturbation, with the spatial distribution being uniform in the rest of the domain.

A decrease in $\varepsilon$ leads to the emergence of the OMZ. It first appears at the position of the initial perturbation (i.e. near the centre of the domain); see the bottom of Fig. 11b,c. At a later time, it breaks to several patches that fast spread over the entire domain. The spatiotemporal dynamics is apparently chaotic for $\varepsilon =0.5$ but becomes more regular for $\varepsilon =0.25$, cf. Fig. 11c,g.

A further decrease in $\varepsilon$ below a certain critical value results in a regime shift, e.g. see Fig. 11d,h obtained for $\varepsilon =0.2$. In this case, a large OMZ is formed at an early stage of system’s dynamics (see the bottom of Fig. 11d). However, after a relatively short time the oxygen concentration fast drops to zero over the entire domain: the global anoxia occurs accompanied by the plankton extinction.

**Fig. 12:**
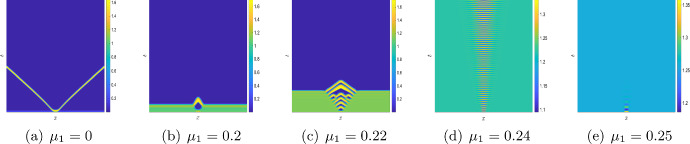
**a**–**e** Transition of spatial distribution of oxygen for the parameter values fixed at (5) and $\mu _2=0.1575,\,D=1$ and $\varepsilon =1$ and for different values of $\mu _1.$

**Fig. 13:**
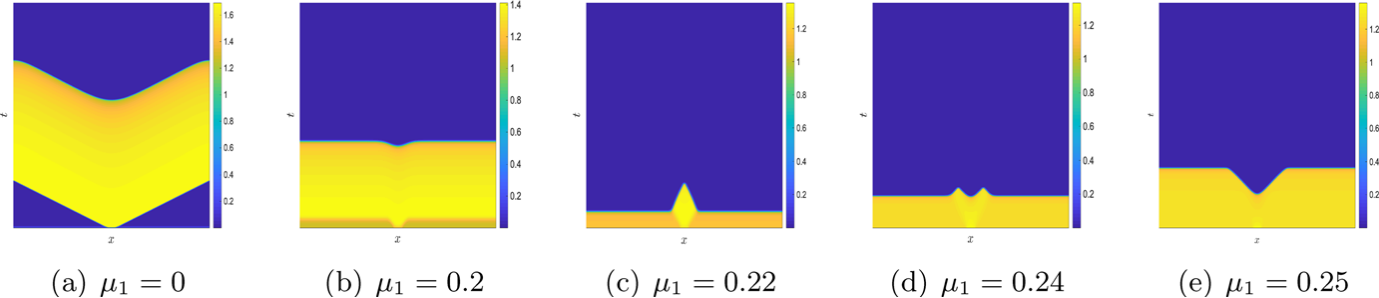
**a**–**e** Transition of spatial distribution of oxygen for the parameter values fixed at (5) and $\mu _2=0.1575,\,D=1$ and $\varepsilon =0.01$ and for different values of $\mu _1.$

We now study the impact of the mortality rates on the system dynamics in Figs. 12 and 13. For $\mu _2=0,$ the system does not exhibit any stationary Turing patterns. However, with the inclusion of the density dependent mortality $(\mu _2)$, we are able to show that the system can produce stationary Turing pattern, even when $\mu _1=0$ (cf. Figure 8). To further study the interplay between the linear mortality rate and the timescale separation, we simulate the system (14) by keeping $\mu _2,\,D$ and $\varepsilon$ fixed along with the parameters (5). In Fig. 12 we show the transition from an extinction state to a steady state with the gradual increase of $\mu _1$ for $\varepsilon =1.$ However, in Fig. 13, we obtain that for sufficiently small values of $\varepsilon$ (that is, for $\varepsilon =0.01$), the OMZ formed at the early stages spreads throughout the domain and the oxygen concentration drops to zero for the increasing values of $\mu _1.$

## Discussion and conclusions

Over the last few decades, there has been growing evidence of a decline of the dissolved oxygen concentration in the ocean (Breitburg et al. 2018; Schmidtko et al. 2017). This has not only been recognized as a catastrophic threat to the marine ecosystems (Wishner et al. 2018) but also as a potential threat to mankind (Martin et al. 2017) and to terrestrial ecosystems, as marine phytoplankton contributes about 70% to the total atmospheric oxygen. Any significant decline in the global phytoplankton abundance and/or a decrease in the oxygen production rate in phytoplankton photosynthesis will inevitably lead to a decline in the global stock of the atmospheric oxygen (Petrovskii et al. 2017; Petrovskii 2021). Thus, marine ecosystems, phytoplankton in particular, play a crucial role in maintaining the habitable Earth (Sudakow et al. 2022).

In spite of the apparent importance of the above issues, mathematical models addressing the change in the oxygen concentration as a component of the coupled phytoplankton–oxygen dynamics are rare in the literature. As one exception, a generic three-component oxygen-phyto-zooplankton model was developed in Sekerci and Petrovskii (2015a) (and further investigated in Petrovskii et al. (2017); Sekerci and Petrovskii (2018, 2018b)). It has been shown that the formation of areas with a low oxygen concentration (i.e. OMZs) is in fact an inherent property of the self-organised plankton–oxygen spatiotemporal dynamics, but it can be exacerbated by the effect of global warming, potentially leading to global anoxia.

The model developed in Sekerci and Petrovskii (2015a); Petrovskii et al. (2017), however, missed several important features of the marine ecosystem’s dynamics, hence making the prediction of emerging global anoxia somewhat questionable. In this paper, we have considered a nontrivial extension of the original model that includes factors such as zooplankton inherent competition, cannibalism and/or the effect of zooplankton’s consumers from upper trophic layers, e.g. fish. Another important factor is the existence of different timescales for phyto- and zooplankton growth, as the latter is usually much slower than the former.

The properties of the extended model have been analysed in much detail using a combination of analytical and numerical tools. We first consider a non-spatial version of the model described by a system of three nonlinear ODEs (for oxygen, phytoplankton, and zooplankton, respectively) to study the variation in oxygen level and plankton densities over time. In addition to the results earlier obtained in Sekerci and Petrovskii (2015a, 2015b), we have shown that the number of coexisting steady states depends both on the linear mortality rate ($\mu _1$) and the rate of zooplankton intraspecific competition/consumption (quantified by coefficient $\mu _2$). For $\mu _1=0,$ there exists two feasible coexisting steady states where the lower oxygen level is always unstable, and the higher state changes its stability with increasing $\mu _2.$ Whereas for $\mu _1\ne 0,$ we obtain a unique feasible steady state which changes its stability from unstable to stable for a strong intraspecific competition. Along with this, the extinction state is always stable. We also found that an increase in the rates of zooplankton linear mortality ($\mu _1$) and nonlinear mortality ($\mu _2$) leads to an increase in the oxygen abundance.

In order to better understand the relative importance of the linear and nonlinear mortality and their effect on the temporal dynamics of the system, we have considered three cases: (a) $\mu _1\ne 0,\mu _2=0,$ (b) $\mu _1=0,\mu _2\ne 0,$ and (c) $\mu _1\ne 0,\mu _2\ne 0$. Because of the complexity of the system, this has mostly been done through numerical simulations. For case (a), the unique steady state is stable for higher values of $\mu _1,$ and it loses its stability through supercritical Hopf bifurcation. A stable cycle then emerges, fast increasing its size within a small interval of $\mu _1.$ Beyond that, the system cannot further withstand an increase in the amplitude of the cycle leading to complete collapse (see Fig. 2a). However, for case (b), the Hopf bifurcation is subcritical, and the system converges to stable steady state for higher values of $\mu _2.$ In this case, an unstable cycle is formed (see Fig. 2b). Case (c) is a combination of the above two cases. Here, in a very narrow domain, the system exhibits tri-stability, with a stable steady state, a stable cycle, and the extinction state. The two cycles appear through saddle-node bifurcation of limit cycle, and the disappearance of the unstable cycle is through subcritical Hopf bifurcation, and that of the outer stable cycle is through heteroclinic bifurcation (see Fig. 2c).

Having analysed the effect of different timescales (cf. “slow-fast system”), we obtain the critical manifold of the slow-fast system by collecting all the equilibrium points of the fast subsystem (8). The critical manifold is then analyzed by investigating the bifurcations occurring in the fast subsystem. In this regard, we obtain that the saddle-node bifurcation point of the fast subsystem corresponds to the fold point of the slow-fast system (cf. Figs. 3 and 4). Small stable canard cycles are obtained when the equilibrium point of the slow-fast system lies in a small neighborhood of the fold point. If the system is pushed far beyond the fold point (e.g. by the choice of the initial conditions), the dynamics will eventually lead to plankton extinction and oxygen depletion, although the extinction/anoxia can be preceded by a long period of oscillations (cf. Fig. 7b). However, as $\varepsilon \rightarrow 0$, the number of oscillations decreases and the system shifts from oscillatory to non-oscillatory (cf. Fig. 7c–f). Further a decrease in the nonlinear mortality rate $\mu _2$ below a certain critical value first destabilises the coexistence steady state resulting in oscillatory dynamics (see Fig. 6). A further decrease (below another critical value) leads to the canard explosion. However, no relaxation oscillations emerge (see Theorem 4.1); instead, the system’s trajectory goes to the origin, which obviously corresponds to the extinctions and anoxia.

We then considered the spatially explicit system to study the distribution and spatiotemporal dynamics of oxygen and plankton. The spatially explicit model consists of three reaction-diffusion equations where the diffusion terms account for the effect of lateral turbulent mixing for the dissolved oxygen and phytoplankton and for the combined effect of turbulence and self-motion for zooplankton. Note that, because of the latter, zooplankton diffusivity can be expected to differ from that of phytoplankton and oxygen. Moreover, it can differ significantly. The interplay between the ordinary Fickian diffusion (in our case “biodiffusion” resulting from zooplankton random movement) and the turbulent mixing is known to be highly nonlinear (Monin and Yaglom 1971, 1975). The ordinary diffusion, although itself often being orders of magnitude less intensive than the turbulent mixing, accelerates the turbulent diffusion significantly (Monin and Yaglom 1971, 1975). In turn, for the diffusivity ratio being greater than one, the model can exhibit pattern formation due to the Turing instability; see Sects. 5.1 and 5.2. However, the earlier works in the direction (Sekerci and Petrovskii 2015a; Petrovskii et al. 2017; Sekerci and Petrovskii 2018, 2018b) does not exhibit stationary Turing pattern.

Due to its mathematical complexity, the spatially explicit reaction-diffusion model is not analytically tractable. Therefore, we have investigated its properties through extensive numerical simulations, with a special attention to regimes that result in the formation of patterns containing areas with low oxygen level and/or regimes resulting in global oxygen depletion. Using the initial condition as a localised perturbation of the spatially uniform steady state, we have obtained that the system dynamics typically lead to the formation of strongly heterogeneous spatial distribution that includes one or several areas (patches) with a very low oxygen level, which we interpret as the formation of OMZ. Interestingly, the patterns emerge both in and outside of the Turing domain and hence, for different parameter values (e.g. the diffusivity ratio being larger or smaller than one) can be attributed to different dynamical mechanisms, i.e. Turing or non-Turing. Except for some rare cases (cf. Fig. 8b), the OMZ formed at an early stage of the system dynamics fast spreads over the entire domain, often generating multiple patches, e.g. see Figs. 8a,c, 10a–c and 11b–d, the size and number of the emerging smaller OMZs varying with the parameter values.

The spread of the emerging pattern (a mixture of patches with high and low oxygen level) can lead to a different outcome. It can result in a self-sustained pattern, which, in the large time limit, can be stationary (cf. Figs. 8c and 10a) or dynamic (Fig. 10b). Alternatively, it may eventually lead to an unsustainable pattern – a regime shift—when, after a certain time, the oxygen concentration fast drops to very small values over the entire domain (cf. Figs. 8a, 10c and 11d). Arguably, this may be interpreted as the onset of the global anoxia.

Note that there is a subtle interplay between the zooplankton linear mortality rate $\mu _1$ and the difference in the timescales. In the special case of the same timescales ($\varepsilon =1$), the initial perturbation at the center of the domain leads to the formation of deoxygenated patch (OMZ), and the system eventually goes to extinction till $\mu _2=0.22.$ However, for a higher value of $\mu _2=0.24,$ it leads to small fluctuations in the oxygen level and further for $\mu _2=0.25,$ the system settles down to a steady state (cf. Fig 12).

However, for $\varepsilon$ sufficiently small, and with a further increase in the timescale separation (i.e. for a smaller $\varepsilon$), the linear mortality rate of the zooplankton makes the system less sustainable. For $\varepsilon =0.01,$ and keeping $\mu _2=0.1575$ and $D=1$ fixed, the global anoxia occurs for all the values of $\mu _2$ (cf. Fig. 13).

For a fixed value of $\mu _1$, an increase in the timescale separation alone makes the dynamics less sustainable. A decrease in $\varepsilon$ tends to lead to a larger size of the initially formed OMZ; e.g. see Fig. 11b–d, eventually resulting in global anoxia and extinctions when $\varepsilon$ becomes sufficiently small. That happens both for $\mu _1=0$ and $\mu _1>0$, cf. Figs. 10c and 11d, although the succession of spatial patterns preceding the onset of anoxia is different between the two cases.

Apparently, our study leaves open questions. Firstly, recall that our model is conceptual; it only takes into account the interaction between oxygen and plankton but not with other components of the complicated marine food web. It has been shown in Petrovskii et al. (2017) that, in case of a trophic chain, the effect of higher trophic levels only makes the regime shift—the catastrophe of oxygen depletion and plankton extinction—more likely as the three-component model (1) provides an upper bound for a longer trophic chain. An open question however remains as to how the dynamics may change in case of a web rather than chain, for instance to account for effect of bacteria or detritus. Secondly, the description of turbulent mixing as the turbulent diffusion is somewhat simplistic; in particular, it completely disregards the fact that the turbulent mixing is multiscale and nonlocal (Monin and Yaglom 1971, 1975). Although the model with the turbulent diffusion is arguably a sensible first step, a more advanced approach should involve a more realistic description of turbulence. These issues will become a focus of future work.

## Data Availability

This paper has no data.

## Appendix

We consider a compartment-type model of the plankton–oxygen dynamics where the three constituting elements or ‘boxes’ are oxygen, phytoplankton and zooplankton (Sekerci and Petrovskii 2015a). Phytoplankton produces oxygen in daytime but consume it during the night. Zooplankton feeds on phytoplankton, hence controlling its abundance, and also uses oxygen for breathing. In the non-spatial case, which is relevant in the case of a well-mixed ecosystem, the corresponding model is given by the following equations:

24 $$\begin{aligned} \frac{dc}{dt}= & {} Af(c)u - u_{r}(c,u) - v_{r}(c,v) - mc, \end{aligned}$$

25 $$\begin{aligned} \frac{du}{dt}= & {} g(c,u) - e(u,v)-\sigma u, \end{aligned}$$

26 $$\begin{aligned} \frac{dv}{dt}= & {} \kappa (c)e(u,v) - M(v), \end{aligned}$$

where *c* is the concentration of oxygen at time *t*, *u* and *v* are the densities of phytoplankton and zooplankton, respectively. The first term in the right-hand side of Eq. (24) describes the oxygen production in photosynthesis (by phytoplankton), the second and third terms describe, respectively, the oxygen consumption by phyto- and zooplankton, the last term accounts for the loss of oxygen in other biochemical processes in the sea water that are not included into the model explicitly. In Eq. (25), functions *g* and *e* describe, respectively, the phytoplankton growth and its loss due to predation by zooplankton; the linear term accounts for the phytoplankton natural mortality. In Eq. (26), the food conversion efficiency $\kappa$ is assumed to depend on the oxygen level, as anoxic conditions (low oxygen concentration) are known to disrupt normal feeding of marine fauna (Riedel et al. 2008; Mosch et al. 2012). The last term in Eq. (26) describes zooplankton mortality; here we consider it in the following form:

27 $$\begin{aligned} M(v)= & {} \mu _1 v +\mu _2 v^2, \end{aligned}$$

where the linear term describes the zooplankton natural mortality and the square term may arise for a variety of reasons, for instance, to account for ‘cannibalism’ or for the effect of zooplankton consumption by higher trophic levels (Truscott and Brindley 1994a). Considering appropriate parameterisation of functions *f*, $u_r$, $v_r$, *g*, *e* and $\kappa$ (for details, please see (Sekerci and Petrovskii 2015a)), Eqs. (24–26) takes the following more specific form:

28 $$\begin{aligned} \frac{dc}{dt}= & {} \frac{Ac_0u}{c+c_0} - \frac{\delta uc}{c+c_2}- \frac{\nu cv}{c+c_3} - mc, \end{aligned}$$

29 $$\begin{aligned} \frac{du}{dt}= & {} \left( \frac{B c}{c+c_1} - \gamma u \right) u - \frac{\beta uv}{u+h} - \sigma u, \end{aligned}$$

30 $$\begin{aligned} \frac{dv}{dt}= & {} \frac{\eta c^2}{c^2+{c_4}^2}~\frac{\beta uv}{u+h} - \mu _1 v - \mu _2 v^2, \end{aligned}$$

where all parameters are non-negative. Biologically relevant values of parameters in the model (28–30) are difficult to estimate but some of them can be found in the literature. In particular, Kumar et al. (1991) estimated the maximum per capita grazing rate by zooplankton as $\beta \approx 0.02$ hour^-1^ $=0.48$ day^-1^, while Li at al. (2011) estimated it as $\beta \approx 1$ day^-1^, hence providing the number of the same order of magnitude. Note that, due to its place in Eq. (30), the product $\eta \beta$ is the maximum (exponential) growth rate which is reached in the limit of large *c* and large *u*. For zooplankton, the duplication time is known to be between 60–90 days (Buesa 2019); correspondingly, we obtain that $\eta \beta \approx 0.01$ day^-1^.

Estimates of the linear zooplankton mortality rate yield the value on the order of $10^{-2}$ day^-1^; in particular, $\mu _1\approx 0.012$ day^-1^ in Kumar et al. (1991) and $\mu _1\approx 0.015$ day^-1^ in Dubovskaya et al. (2015). For the phytoplankton mortality rate, it can vary in a broad range between less than 0.01 day^-1^ and more than 1 day^-1^ (Marbá et al. 2007), while Obayashi and Tanoue (2002) provide somewhat narrower range as $0.15<\sigma <0.88$ day^-1^.

The zooplankton nonlinear mortality rate was estimated by Cruz et al. (2021) as $\mu _2=0.025$ and $\mu _2=0.05$ density^-1^day^-1^ for small and large zooplankton, respectively (where the zooplankton density was quantified by the nitrogen content, i.e. as [density]=mmol N meter^-3^). Since our model does not distinguish between small and large zooplankton, we assume that the ‘true’ value of $\mu _2$ is somewhere between 0.025 and 0.05. For parameter *m*, we are not aware about any direct estimations of its value in the literature. However, one can try to estimate it indirectly. It has been reported by several empirical studies that under warm weather condition with little or no wind, anoxic conditions in the sea water can develop over 5–10 days. Correspondingly, it leads to a rough estimate of the de-oxygenation rate as $0.1<m<0.2$ day^-1^.

It is convenient to introduce dimensionless variables. Let us consider

31 $$\begin{aligned}{} & {} t'=t m, \qquad c'=\frac{c}{c_0}, \qquad u'=\frac{\gamma u}{m}, \qquad v'=\frac{\beta v}{m}, \end{aligned}$$

and the new (dimensionless) parameters as

32 $$\begin{aligned}&\hat{B}=\frac{B}{m}, \quad \hat{A}=\frac{A}{c_0\gamma }, \quad \hat{\delta }=\frac{\delta m}{c_0\gamma }, \quad \hat{\nu }=\frac{\nu m}{\beta c_0}, \quad \hat{\mu _1}=\frac{\mu _1}{m}, \quad \hat{\mu _2}=\frac{\mu _2}{\beta }, \end{aligned}$$

33 $$\begin{aligned}&\hat{\sigma }=\frac{\sigma }{m}, \quad \hat{h}=\frac{\gamma h}{m}, \quad \hat{\eta }=\frac{\eta \beta }{m}, \quad \text{ and }\quad \hat{c_i}=\frac{c_i}{c_0}~~\text{ where }~~i=1,2,3,4. \end{aligned}$$

Equations (28–30) then turn into the following:

34 $$\begin{aligned} \frac{dc}{dt}= & {} \frac{Au}{c+1} - \frac{\delta uc}{c+c_2}- \frac{\nu cv}{c+c_3} - c, \end{aligned}$$

35 $$\begin{aligned} \frac{du}{dt}= & {} \left( \frac{B c}{c+c_1} - u \right) u - \frac{u v}{u+h} - \sigma u, \end{aligned}$$

36 $$\begin{aligned} \frac{dv}{dt}= & {} \frac{\eta c^2}{c^2+{c_4}^2}~\frac{uv}{u+h} - \mu _1 v - \mu _2 v^2, \end{aligned}$$

now in terms of dimensionless variables and parameters, where primes and hats are omitted for the notations simplicity. Note that meaningful range of dimensionless parameters is, generally speaking, different from that of the original (dimensional) parameters. We readily notice that, for the ‘realistic’ parameter values (as above), the dimensionless parameters determining zooplankton dynamics are typically rather small being in the corresponding ranges as

37 $$\begin{aligned} \hat{\mu _1}=\frac{\mu _1}{m}\approx 0.05\text{-- }0.1, \quad \hat{\mu _2}=\frac{\mu _2}{\beta }\approx 0.025\text{-- }0.1, \quad \hat{\eta }=\frac{\eta \beta }{m}\approx 0.05\text{-- }0.1. \end{aligned}$$

For mathematical analysis of the system, it is convenient to take into account this overall ‘smallness’ of the zooplankton parameters by introducing a common factor, say $\varepsilon$, so that $\varepsilon \ll 1$. Equation (36) then turns into the following:

38 $$\begin{aligned} \frac{dv}{dt}= & {} \varepsilon \left( \frac{\tilde{\eta } c^2}{c^2+{c_4}^2}~\frac{uv}{u+h} - \tilde{\mu _1} v - \tilde{\mu _2} v^2\right) , \end{aligned}$$

where new parameters $\tilde{\eta }=\eta /\varepsilon$, $\tilde{\mu _1}=\mu _1/\varepsilon$ and $\tilde{\mu _2}=\mu _2/\varepsilon$ are on the order of unity.
